# *Leuconostoc mesenteroides* and *Liquorilactobacillus mali* strains, isolated from Algerian food products, are producers of the postbiotic compounds dextran, oligosaccharides and mannitol

**DOI:** 10.1007/s11274-024-03913-3

**Published:** 2024-02-29

**Authors:** Kenza Zarour, Ahmed Fouad Zeid, Mari Luz Mohedano, Alicia Prieto, Mebrouk Kihal, Paloma López

**Affiliations:** 1 Departamento de Biotecnología Microbiana y de Plantas, Centro de Investigaciones Biológicas Margarita Salas (CIB, CSIC), 28040 Madrid, Spain; 2 Laboratoire de Microbiologie Appliquée, Faculté des Sciences de la Nature et de la Vie, Université Oran 1 Ahmed Ben Bella, Es Senia, 31100 Oran, Algeria

**Keywords:** Dextran, Dextranase, Dextransucrase, Lactic acid bacteria, Mannitol, Riboflavin

## Abstract

Six lactic acid bacteria (LAB) isolated from Algerian sheep’s milk, traditional butter, date palm sap and barley, which produce dextran, mannitol, oligosaccharides and vitamin B_2_ have been characterized. They were identified as *Leuconostoc mesenteroides* (A4X, Z36P, B12 and O9) and *Liquorilactobacillus mali* (BR201 and FR123). Their exopolysaccharides synthesized from sucrose by dextransucrase (Dsr) were characterized as dextrans with (1,6)-d-glucopyranose units in the main backbone and branched at positions *O*-4, *O*-2 and/or *O*-3, with d-glucopyranose units in the side chain. A4X was the best dextran producer (4.5 g/L), while the other strains synthesized 2.1–2.7 g/L. Zymograms revealed that *L. mali* strains have a single Dsr with a molecular weight (Mw) of ~ 145 kDa, while the *Lc. mesenteroides* possess one or two enzymes with 170–211 kDa Mw. As far as we know, this is the first detection of *L. mali* Dsr. Analysis of metabolic fluxes from sucrose revealed that the six LAB produced mannitol (~ 12 g/L). The co-addition of maltose–sucrose resulted in the production of panose (up to 37.53 mM), an oligosaccharide known for its prebiotic effect. A4X, Z36P and B12 showed dextranase hydrolytic enzymatic activity and were able to produce another trisaccharide, maltotriose, which is the first instance of a dextranase activity encoded by *Lc. mesenteroides* strains. Furthermore, B12 and O9 grew in the absence of riboflavin (vitamin B_2_) and synthesized this vitamin, in a defined medium at the level of ~ 220 μg/L. Therefore, these LAB, especially *Lc. mesenteroides* B12, are good candidates for the development of new fermented food biofortified with functional compounds.

**Supplementary Information:**

The online version contains supplementary material available at 10.1007/s11274-024-03913-3.

## Introduction

In Algeria, the consumption of natural traditional foods holds a central place in daily life and culture. Among them, sheep’s milk plays a significant role in many preparations, notably in Algerian cheese known as *Jben*. (Benheddi and Hellal 2019). This type of milk, along with cow’s milk, often forms the basis for traditional butter, and its artisanal production method strengthens the connection between tradition and local practices (Boussekine et al. 2022). Also, barley and the sap of the date palm, known as *Lagmi*, are also fundamental and highly nutritious ingredients in many regions of Algeria, particularly in the south (Ben Thabet et al. 2009). Consequently, the knowledge of these traditional foods bacterial microbiota, and their characterization, could increase their functionality and help in the future to use these microorganisms for the development of new functional food.

In this context, lactic acid bacteria (LAB), present in these traditional foods, would be a good target for isolation, since many LAB and their nontoxic metabolites have been approved by the United States Food and Drug Administration and the European Food Safety Agency as “generally recognized as safe” with a “qualified presumption of safety” (Russo et al. 2017). LAB are present in many ecological niches, although their abundance and density vary according to the growth environment (George et al. 2018). Thus, they are naturally present in food matrices and products, the gastrointestinal tract of humans and other animals, soil and water (Wang et al. 2022b). In addition, numerous LAB species can produce various metabolites which are classified as postbiotics (Nataraj et al. 2020), such as lactic acid, antimicrobial substances, aroma compounds and exopolysaccharides (EPS), which are involved in anti-bacterial, anti-fungal, anti-biofilm and anti-viral activities (Kavitake et al. 2023) and contribute positively to functional food manufacture (Mora-Villalobos et al. 2020). In fact, many LAB belonging to *Leuconostoc*, *Weissella* and *Lactobacillus* genera, can produce EPS, specifically dextrans. These homopolysaccharides are α-glucans consisting of a linear backbone of d-glucopyranosyl units with α-(1,6) linkages in the main chain and variable percentages of α-(1,4), α-(1,3), or α-(1,2) ramifications (Korcz and Varga 2021). Dextrans are biosynthesized by dextransucrases (Dsr), which belong to the glycosyl hydrolases (GH) family 70. These enzymes hydrolyse sucrose generating fructose and glucose molecules, and also catalyse the transfer of a glucose molecule onto a growing chain of α-glycosidically linked polymers (Soumya and Nampoothiri 2021). The α-glucans have several applications in the pharmaceutical and cosmetic industries. Furthermore, their utilization in food industry is wide, because, besides being a safe food additive, they can act as gelling, emulsifying, stabilizing, water-binding, and viscosifying agents. Also, they play significant roles in the organoleptic properties of food (Daba et al. 2021; Pérez-Ramos et al. 2015). In addition, we have previously shown that high molecular weight (Mw) dextrans have antiviral and immunomodulatory activities (Nácher-Vázquez et al. 2015; Zarour et al. 2017). Recently, the baking industry, mainly in relation to the manufacture of gluten-free products, has been interested in the use of EPS-producing bacteria (Korcz and Varga 2021).

Besides the sucrose reaction, it has been shown that the Dsr of some strains belonging to the *Lactobacillus*, *Leuconostoc*, *Weissella* or *Streptococcus* genera are able to catalyse an acceptor reaction, when other carbohydrates, in addition to sucrose, are present. The reaction allows the production of oligosaccharides such as the glucotrisaccharides maltotriose and panose, which are known for their prebiotic properties, promoting the growth of beneficial gut bacteria and potentially offering health benefits (Bivolarski et al. 2018).

In addition to biopolymer production, LAB can produce mannitol through fermentation. This production can be very efficient from fructose by the action of mannitol dehydrogenase, in the case of heterofermentative bacteria such as some lactobacilli and strains belonging to the *Leuconostoc* and *Oenococcus* genera (Martínez-Miranda et al. 2022). Mannitol is the main sugar alcohol or polyol synthesized by LAB and it has gained significant attention in the food industry for its various functional properties and health benefits, and is currently used as: (i) sugar substitute in food formulations; (ii) texturizing agent; (iii) humectant; (iv) cryoprotectant during the freeze-drying processes and (v) ingredient in low-glycemic foods (Ding and Yang 2021; Gok et al. 2020; Xu et al. 2022; Zhu et al. 2023).

Furthermore, some LAB can produce, other secondary metabolites such as riboflavin (vitamin B_2_) during the fermentation processes, which makes them attractive hosts for vitamin production for *in situ* food biofortification (Zhu et al. 2020), to counteract the vitamin deficiency linked to malnutrition, insufficient food intake and unbalanced diets (Gaspar et al. 2013).

The use of postbiotic-producing LAB in food and human health industries seems to be a more natural and economical alternative than fortification with molecules chemically synthetized (Chadare et al. 2019). In this context, this study is focused on the isolation, from butter, goat’s milk and plant ecological niches, of new LAB strains with health promoting properties, especially production of dextran, mannitol, prebiotics oligosaccharides (panose, maltotriose) and riboflavin, in order to develop Algerian functional biofortified food.

## Materials and methods

### Bacterial isolation and culture conditions

The six LAB strains studied in this work were isolated from four Algerian ecosystems: A4X, FR123 and Z36P from fresh sap of date palm (*Phœnix dactylifera* L.) of southern Algeria, precisely from the Ouargla region. O9 from fresh barley collected in Oran region, and B12 and BR201 from traditional butter and sheep’s milk, respectively, which have been sampled from the Oran region. These bacteria were isolated after growth in MRS medium without dextrose (Pronadisa, Spain) supplemented with 2% sucrose (MRSS) at 30 °C for 72 h, in order to detect LAB producing EPS. For long-term storage at − 80 °C, MRS medium supplemented with 20% (v/v) glycerol was used. For testing of EPS and riboflavin production, we used: the riboflavin assay medium lacking riboflavin (BD Difco™, France), which contains 2% glucose (RAMG), the RAMG supplemented with 2% sucrose (RAMGS) and/or supplemented with riboflavin at 1 µg/mL (RAMGSR).

### Phenotypic characterization

The presumptive LAB strains selected as Gram-positive and catalase-negative bacteria and generating ropy colonies on MRSS agar, were identified using the following physiological and biochemical tests: (i) gas production; (ii) hydrolysis of arginine; (iii) growth in MRS supplemented with 2% glucose (MRSG) at different temperatures (4 °C, 15 °C, 37 °C and 45 °C), in the presence of 3.0% or 6.5% NaCl, and at different pH values (4.0 and 8.0); (iv) citrate metabolism; (v) acetoin production and (vi) sugar fermentations patterns using API 50 CHL (Biomerieux, France), as previously described (Zarour et al. 2018).

### Genotypic characterization

**Fig. 1:**
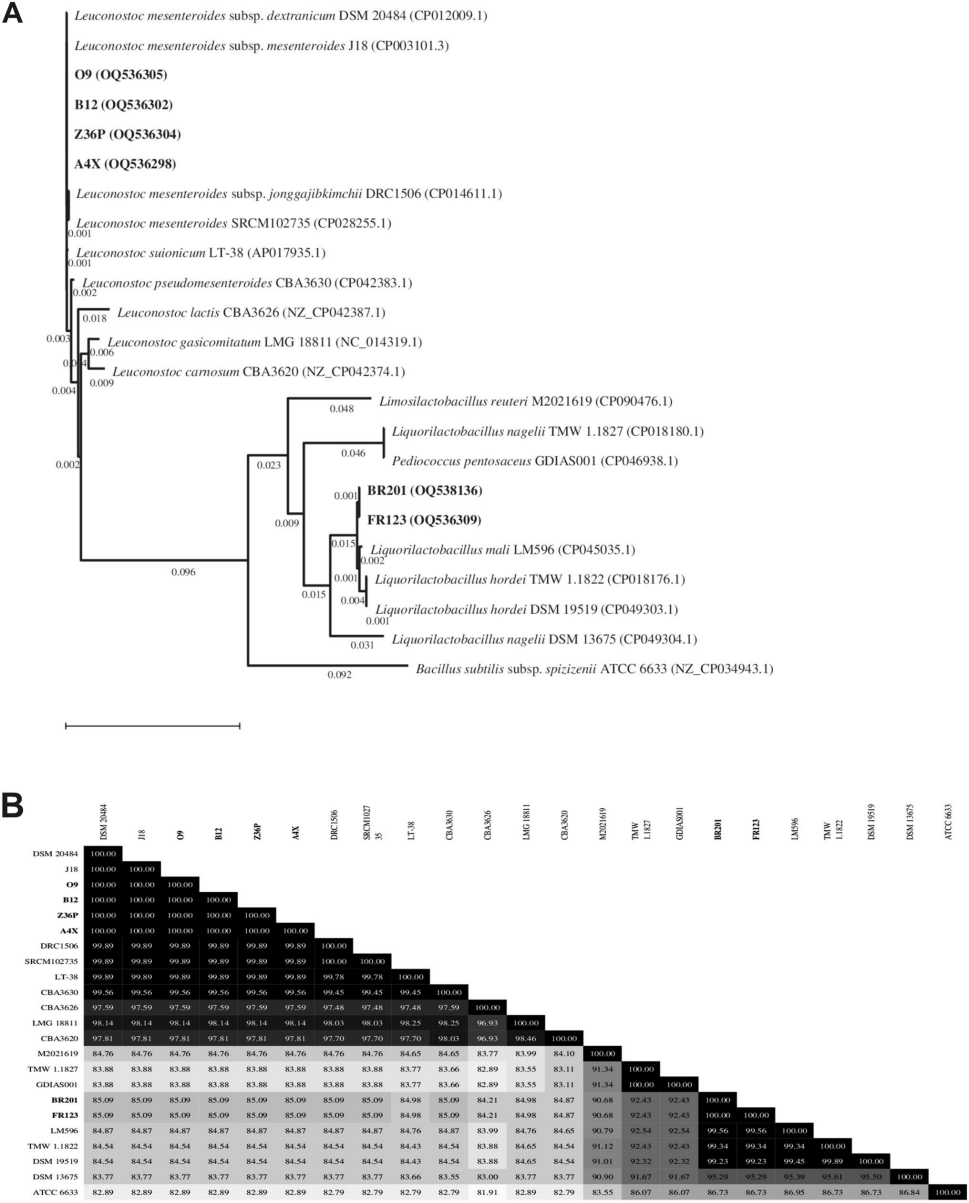
Neighbour joining phylogenetic rooted tree based on the partial sequences of the 16S rRNA coding gene, showing the taxonomic location of the analyzed strains (**A**) and the degree of identity (%) among them (**B**). The evolutionary distances showed in **A** were computed using the Maximum Composite Likelihood method and are in the units of the number of base substitutions per site. The analysis involved 23 nucleotide sequences. There were a total of 912 positions in the final dataset. Accession numbers from GenBank are given in brackets. Evolutionary analyses were conducted with the MegAlign Pro Software (DNASTAR). The taxonomic denomination and the isolation habitat and country of the strains is depicted in Online Resource Table S3

**Fig. 2:**
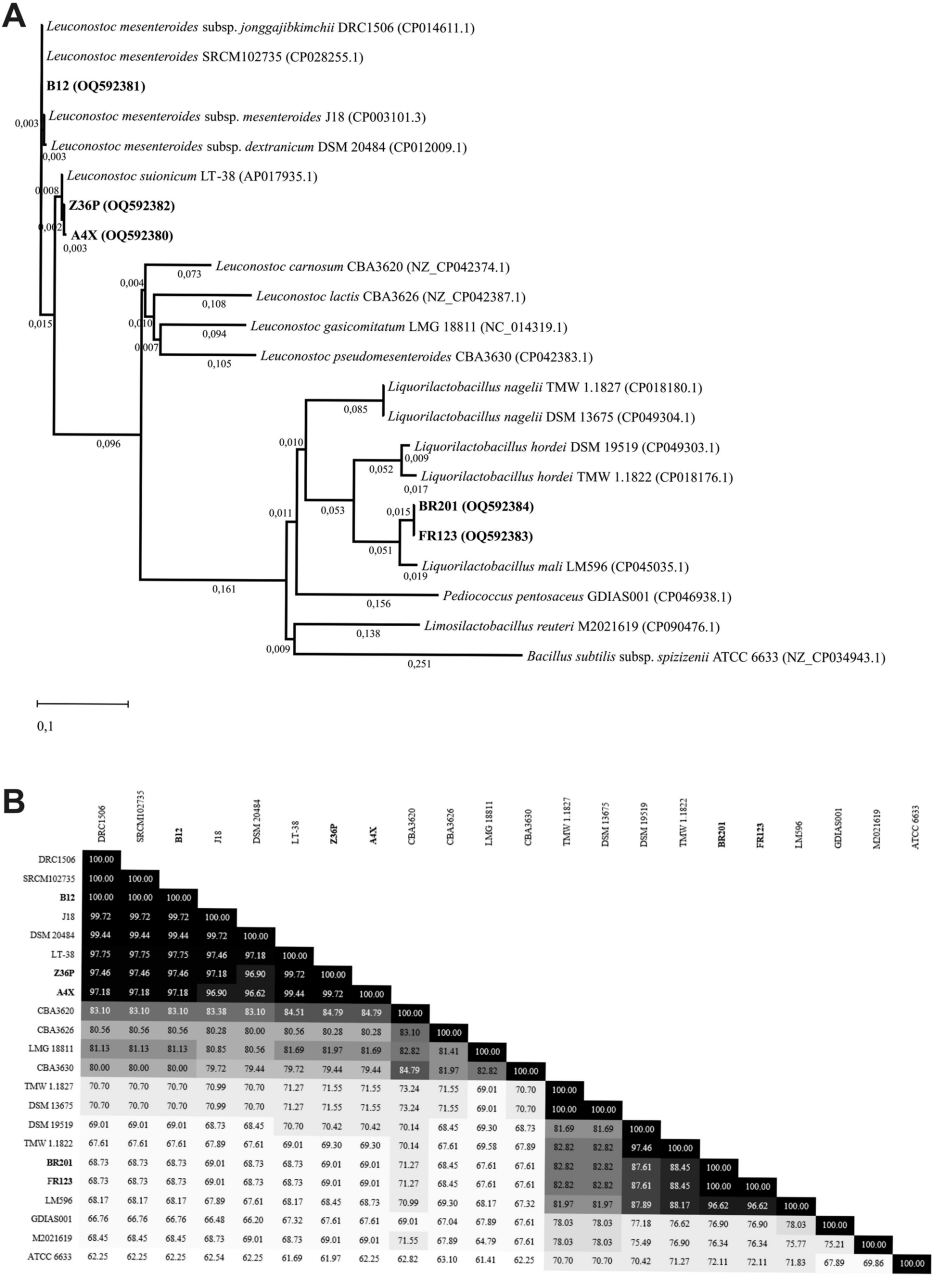
Neighbour joining phylogenetic rooted tree based on the *pheS* gene partial sequences showing the taxonomic location of the analysed strains (**A**) and the degree of identity (%) among them (**B**). The evolutionary analysis was performed as described in legend of Fig. 1. The analysis involved 22 nucleotide sequences. There were a total of 355 positions in the final dataset. The taxonomic denomination and the isolation habitat and country of the strains is depicted in Online Resource Table S4

The amplification and sequencing of the variable regions of the *rrs* (also called *16S rRNA*) and the *pheS* genes of the six isolates were performed by the Sequencing DNA Service at Secugen (Madrid, Spain). The obtained sequences were deposited in the GenBank (see accession numbers below in Figs. 1, 2) and initially pairwise compared with other sequences of the type strains held in the GenBank using the Blast Nucleotide Program from the National Center for Biotechnology Information (NCBI) (Altschul et al. 1990). Then, the MegAlign Pro Software DNASTAR Navegator 17 was used to perform the following analyses of the six Algerian isolates: (i) their sequences and those of the selected type strains were multi-aligned using the ClustalW method (Thompson et al. 1997); (ii) their evolutionary history was inferred using the Neighbor-Joining method (Saitou and Nei 1987) and (iii) their evolutionary distances were computed using the Maximum Composite Likelihood method (Tamura et al. 2004).

### Detection of EPS production by LAB at colony and cellular levels

For phenotypical detection, LAB strains were grown in liquid MRSG medium at 30 °C until reaching an optical density at 600 nm of 1.0 (OD_600nm_ = 1.0). Then, 100 µL of 10^−6^ dilutions of the bacterial cultures were streaked on MRSS- and MRSG-agar plates and incubated at 30 °C for 24 h. The colonies were used to detect EPS location by transmission electron microscopy (TEM) as previously described (Llamas-Arriba et al. 2021). The preparations were examined in a JEOL JEM-1230 electron microscope (JEOL Ltd., Tokyo, Japan) operating at an accelerating voltage of 100 kV.

### Quantification of EPS and riboflavin production

The six LAB were grown in MRSG at 30 °C to OD_600nm_ = 1.0, then, pre-cultures were resuspended in RAMGS (2 mL) or RAMGSR to give OD_600nm_ = 0.1, grown for 24 h at 30 °C and sedimented by centrifugation (9000×*g*, 10 min). After that, the supernatants were analysed to quantify the EPS and riboflavin present in them. The EPS present in the culture supernatants were precipitated with three volumes of cold absolute ethanol, washed twice with 80% (v/v) ethanol and their concentrations were estimated by measurement of the total sugar content by the phenol–sulphuric acid method (Dubois et al. 1956) using a glucose calibration curve. The riboflavin fluorescence was measured upon excitation at a wavelength of 440 nm and detection of emission at a wavelength of 520 nm, as previously described (Mohedano et al. 2019). After measurement of riboflavin fluorescence, the concentration of the vitamin in the samples was calculated using a calibration curve constructed to correlate the fluorescence emitted at 520 nm by solutions containing increasing concentrations of riboflavin dissolved in RAM (Hernández-Alcántara et al. 2022).

### Purification and characterization of EPS

For EPS production, the LAB were grown in MRSS liquid medium as indicated above Then, bacteria were sedimented by centrifugation (7300×*g*, 1 h), and the EPS, present in culture supernatants, were precipitated with cold absolute ethanol (v/v) at 4 °C during 20 h. After centrifugation (7300×*g*, 1 h), the supernatants were removed and the pellets were dried, resuspended in water, dialyzed in a membrane with a 10 kDa cut off for 24 h at room temperature and lyophilized as described previously (Llamas-Arriba et al. 2019; Nacher-Vazquez et al. 2015).

The EPS concentrations were determined, in each step, as detailed above. The potential contaminants (DNA, RNA and proteins) were detected using specific fluorescent staining kits and the Qubit® 2.0 fluorometric detection methods (Thermo Fisher Scientific, Waltham, MA, USA).

The purified biopolymers were characterized as previously described (Notararigo et al. 2013) in order to determine: (i) monosaccharide composition (by gas-chromatography, after acid hydrolysis); (ii) linkage types (by methylation analysis), and (iii) anomeric configuration (by Fourier transform infrared (FT-IR) spectroscopy).

### Influence of glucose and sucrose on LAB growth

The LAB were grown at 30 °C in MRSG until OD_600 nm_ = 1.0. These pre-cultures were sedimented by centrifugation (9300×*g*, 20 min) and resuspended to give an OD_600 nm_ = 0.1 in MRSG or MRSS for analysis of LAB growth in rich medium for detection of bacterial growth. Then, aliquots of each culture (200 µL) were analysed in sterile 96-well optical white w/lid cell culture polystyrene plates (Thermo Fisher Scientific), in triplicate or in duplicate, those resuspended in either rich or defined media, respectively. The cultures were incubated at 30 °C in a Varioskan Flask System (Thermo Fisher Scientific), and their analysis was performed in real time with measurements every 30 min during 16 h at OD_600 nm_. The growth rate (µ) and generation time (G) of the LAB in liquid media was determined as previously described (Widdel 2007).

The observation of cell aggregation was performed in cultures grown in MRSG or MRSS in tubes, after 24 h incubation at 30 °C.

### Influence of glucose, sucrose and maltose on metabolites production by LAB

The six LAB were grown in MRSG or MRSS to an OD_600nm_ = 1.0. Then, the precultures were diluted 1:100 as follow: (i) the MRSG cultures in both MRS supplemented with 2% of glucose (MRSG) and MRSG supplemented with 10% of maltose (MRSGM) and (ii) the MRSS cultures in MRS supplemented with 4% of sucrose alone or in combination with 10% of maltose (MRSSM). Afterwards, the cultures were grown at 30 °C for 24 h, and after centrifugation (9000×*g*, 10 min, 4 °C), the supernatants were analysed by gas chromatography-mass spectrometry (GC-MS) using *myo*-inositol as internal standard, as previously described (Besrour-Aouam et al. 2021).

### Detection in situ of Dsr activity by zymogram and dextranase activity in plates containing dextran blue

Overnight precultures of the LAB were used as inoculum of the MRSG or MRSS medium, grown for 24 h at 30 °C, sedimented by centrifugation (9000×*g*, 10 min) and the supernatants analysed as previously described with some modifications (Besrour-Aouam et al. 2021). Briefly, the procedure was as follows. First, the culture supernatants were loaded onto an 8% SDS-polyacrylamide gel (7.2 × 8.6 cm^2^) with a 5% stacking gel, and subjected to electrophoresis, at room temperature at a constant voltage (100 V), until 1 h after the dye had reached the bottom of the gel. The loading volume of the samples was calculated taking into account the final OD_600 nm_ of the cultures (Online Resource Table S1) to perform a comparative analysis of Dsr concentration in the various supernatants adjusted for the bacterial biomass present in each culture. To renature proteins after the electrophoresis, the gel was washed three times with sodium acetate buffer for 1 h, and incubated overnight in the same buffer supplemented with 10% sucrose for dextran synthesis. Detection of Dsr activities was carried out by staining of the synthesized polymer using periodic acid-Schiff staining. To estimate the Dsr Molecular weight (Mw), the Pre-stained Precision Plus Protein^TM^ (Bio-Rad laboratories) including ten polypeptides in the range of 10–250 kDa was used. The quantification of the bands as well as their sizes were carried out using the Image Lab 6.1 Software (Bio Rad laboratories).

The dextranase activities of the LAB were detected using a previously described method with modifications (Besrour-Aouam et al. 2021; Tamura et al. 2007). The bacteria were grown to OD_600 nm_ = 1.0 and 5 µL of total culture were spotted on MRSG-agar supplemented with 0.4% dextran blue. The plates were incubated at 30 °C up to 20 days. The appearance of a clear halo around spots were observed.

### Statistical analysis

The experiments including analysis of EPS and riboflavin production as well as the analysis of metabolites production from sugars catabolism were regarded as a completely random design. Measures of EPS and metabolites production were analysed with a factorial (two-way) ANOVA, while riboflavin determinations were analysed with a one-way ANOVA. A *p* value of ≤ 0.05 was considered significant. Mean pairwise comparisons were computed with a Tukey's test (α = 0.05) and results are shown with letters; means with the same letter are not significantly different. All analyses were performed with the R software version 4.3.0 (R Core Team 2023).

## Results

### Isolates identification

**Fig. 3:**
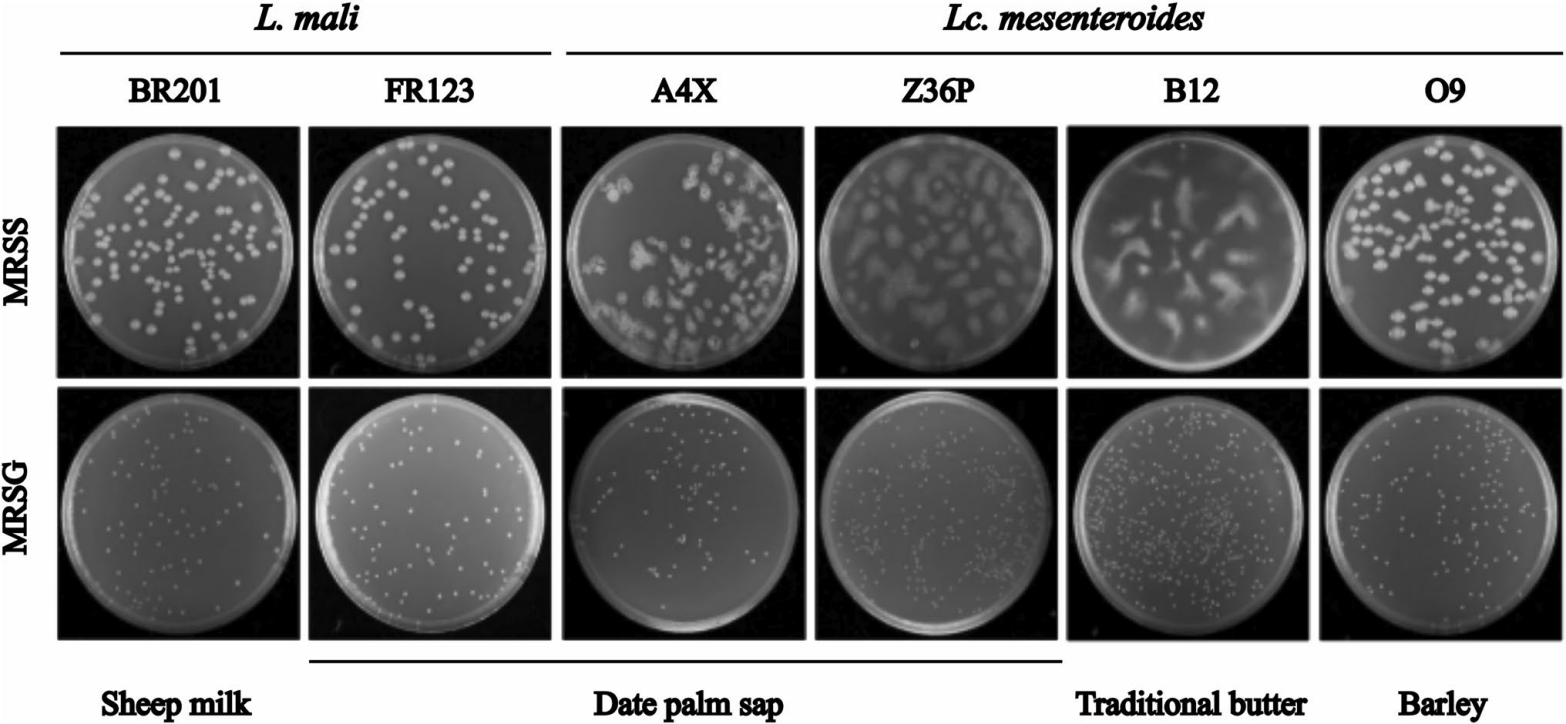
Detection of EPS production by the six LAB at the macroscopic level on solid media. Appearance of bacterial colonies after growth on MRSS-agar or MRSG-agar for 24 h at 30 °C

Gram positive and catalase negative presumptive LAB were isolated from four vegetable and dairy Algerian matrices. Then, to identify bacteria able to synthesize EPS, they were phenotypically tested by plating on MRSS agar, which contains sucrose, the most used method to select LAB strains producing homopolysaccharides. From the screening of fifty strains, six bacteria were selected based on their ropy or mucoid phenotype (see below Fig. 3) and designated A4X, B12, BR201, FR123, O9 and Z36P. Physiological and biochemical tests of the selected isolates were performed according to the procaryotes Bergey’s Manual of Systematic Bacteriology (Vost et al. 2011) in order to position LAB species within their different genera and even at the species level. The results are summarized in Online Resource Tables S2 and S3. The six LAB are unable to hydrolyse arginine, and only five of them coded A4X, B12, Z36P and O9 are able to produce CO_2_, when glucose was the carbon source. They grew at 15 °C, 30 °C and 37 °C but not at 4 °C or 45 °C. Concerning resistance to osmotic stress, all LAB grew in the presence of 3% (w/v) NaCl, and only A4X, BR201 and FR123 withstand 6.5% (w/v) salt exposure. All strains grew in MRSG medium initially adjusted to pH 4.0, but none of them is able to grow in MRSG at pH 8.0. The citrate utilization test is a phenotypic test used for: (i) bacterial classification of bacteria belonging to the species *L. mesenteroides* and (ii) detection of a technological character for the aroma compound production. In this case, the six bacteria are able to metabolize citrate as a precursor of aromatic compounds and A4X, BR201 and FR123 produce acetoin. Moreover, the overall characterization of the six Algerian LAB performed here (Online Resource Table S1), plus the results of their analysis in an apiweb system of API 50 CHL (Online Resource Table S2) allowed to stablish that they probably belong to the *Liquorilactobacillus mali* (BR201 and FR123) and to the *Leuconostoc mesenteroides* (A4X, B12, O9 and Z36P) species.

### Genetic typing of the LAB strains

To confirm the identity of the LAB, they were subjected to molecular identification at the species level and to taxonomical classification by DNA typing, the method most used to classify bacteria. To this end, the partial variable sequence of the coding genes of the 16S rRNA (*rrs*) and of the α-subunit of phenylalanyl-tRNA synthetase (*pheS*) of the LAB was determined, deposited in the GenBank and compared with the bacterial GenBank database. This analysis confirmed that the Algerian LAB were either *L. mali* or *Lc. mesenteroides* strains. In addition, the gene sequences were used to develop phylogenetic trees of the isolates. For this purpose, other sequences of catalogued strains belonging to various species of either *Liquorilactobacillus* or *Leuconostoc* genus were obtained from the GenBank database and subjected to a multi-align comparison together with the sequences of the six isolates and those of the *Bacillus subtilis* ATCC6633 used as a guide as a less related sequence. Subsequently, the phylogenetic trees based on the *rrs* (Fig. 1A) and *pheS* (Fig. 2A) gene sequences, alignments were generated and the homology inferred from them expressed as percentage of identity as depicted in Figs. 1B and 2B, respectively. In addition, the taxonomic name of all the strains as well as the habitat and country in which they were isolated are depicted in Online Resource Tables S3 and S4.

The neighbor-joining tree of the 16S rRNA coding genes provided the following information (Fig. 1), the A4X, Z36P, O9 and B12 Algerian strains isolated from date palm sap barely and traditional butter, respectively, possessed the same DNA sequence in the region of the *rrs* genes analysed. Moreover, the genes of the *Lc. mesenteroides* South Korea strains showed: (i) 100% identity for the genes of the strains DSM 20484 isolated from cheese and J18 isolated from kimchi, belonging to subsp. *dextranicum* and *mesenteroides*, respectively, and (ii) 99.8% identity for the genes of the DRC1506 strain (subsp*. jonggajibkimchii*) isolated from soybean paste.

With regards, to other LAB belonging to several species of the *Leuconostoc* genus (*suionicum*, *pseudomesenteroides, lactis*, *gasicomitatum* and *carnosum*) they carry 16S rRNA coding genes with 99.89–97.81% homology to the genes of the *Lc. mesenteroides* Algerian strains. On the other hand, the *rrs* genes of the Algerian strains BR201, isolated from sheep milk, and FR123, isolated from date palm sap were identical. They also presented 99.56% of similarity with the gene of the *L. mali* LM596 strain isolated from South Korean apple juice, and similarity ranging from 99.34 to 95.29% with strains belonging to species of the *Liquorilactobacillus* genus; *L. hordei* TMW 1.1822 from Germany, *L. hordei* DSM 19519 from Belgium and *L. nagelii* DSM 13675 from USA isolated from water kefir, malted barley and partially fermented wine.

The neighbor-joining tree based on the DNA sequence of the *pheS* genes (Fig. 2) revealed a different classification. The genes of the strains A4X and Z36P, isolated from date palm sap showed 99.72% identity and also very high identity (99.44% and 99.72%, respectively) with that of the *Lc. suionicum* LT-38 strain, isolated in Japan. This reference specie was classified as a subspecies *of Lc. mesenteroides* until 2017. In addition, a higher relatedness (100% identity) was detected between the genes of the B12 strain and those of *Lc. mesenteroides* SRCM102735 and *Lc. mesenteroides* subsp *jonggajibkimchii* DRC1506, isolated from soybean paste and kimchi in South Korea, respectively, as well as a similarity of 99.72% with *Lc. mesenteroides* subsp. *mesenteroides* J18 from South Korean kimchi. In the case of the O9 strain, no amplification was obtained for the *pheS* partial gene and it could not be included in the tree. No changes were detected in the BR201 and FR123 classification compared with those obtained from the *rrs* gene tree. It was also observed for the classification of the bacilli culture collection, that the *rrs* genes of *L. nagelii* TMW 1.1827, isolated from German water kefir, and DSM 13675, obtained from partially fermented wine in USA, were clustered in the same subclade, showing a 100% identity. In the 16S rRNA coding gene tree, they were separated in different subclades with 91.67% of similarity.

### Characterization of EPS produced by LAB

The six Algerian strains generated mucoid colonies on plates containing MRSS agar supplemented with sucrose whereas no production of EPS was detected in the presence of glucose (MRSG medium) (Fig. 3). Furthermore, three types of morphology on MRSS-agar plates were observed: (i) *L. mali* FR123 and BR201 as well as *Lc. mesenter*oides O9 strains presented convex and compact colonies firmly adhered to the agar with persisting consistent gel gum texture; (ii) *Lc. mesenteroides* A4X, Z36P strains showed flat colonies with low consistency and a degraded appearance and (iii) *Lc. mesenteroides* B12 strain display irregular colonies, with a diffused liquid gel aspect even during the first hours of polymers production (Fig. 3).

**Fig. 4:**
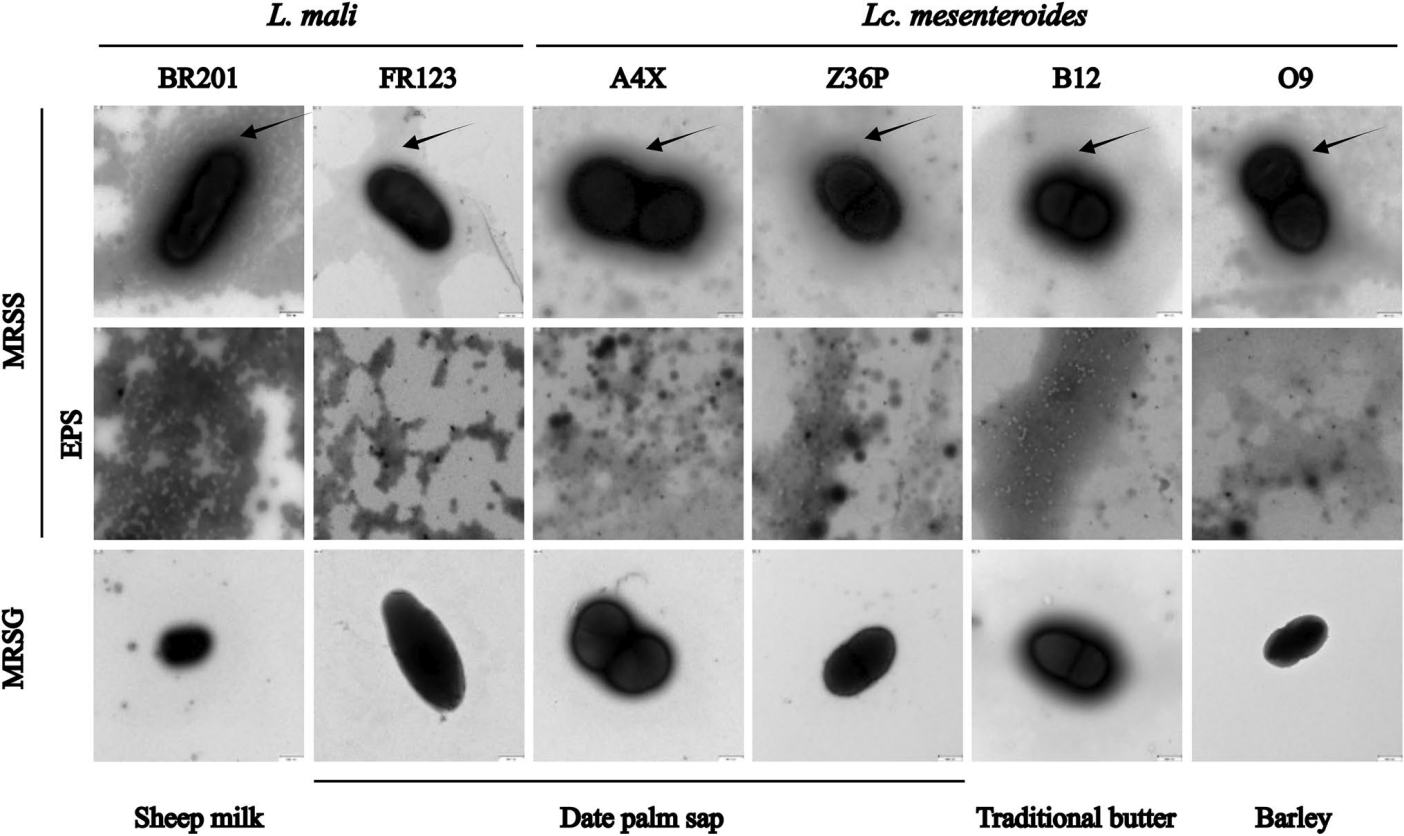
Detection by TEM of EPS production by LAB at microscopic level using colonies grown on solid media. Pictures of either bacterial cells or EPS were taken from samples of LAB colonies after growth at 30 °C for 24 h. The arrows indicate the EPS attached to cells grown in presence of sucrose (MRSS)

To determine the EPS location at the microscopic level, the mucous colonies of the strains were used to be directly analysed by TEM. As shown in Fig. 4, in the samples from MRSS-agar plates, the biopolymers synthesized by the LAB were surrounding the bacterial cells and most of them were not attached. Moreover, EPS was not observed in the TEM analysis for all strains grown on MRSG-agar plates (Fig. 4).

In order to characterize the EPS produced by the Algerian LAB, and to reach a higher bacterial biomass, the six strains were grown in RAMGSR (RAMGS supplemented with riboflavin) liquid medium, and the EPS present in the culture supernatants were purified by precipitation with ethanol (1:1) followed by a dialysis step. Table 1 shows the concentration of the EPS produced by the LAB and the level of contamination prior to, and after purification. The four *Lc. mesenteroides* A4X, Z36P, B12 and O9 strains produced, respectively, 3.90 mg/mL, 2.42 mg/mL, 2.65 mg/mL and 2.12 mg/mL of EPS, with high purity even in culture supernatants, which varies between 93.4 to 98.6%. The polymers, in this step, were free of RNA, with low contamination of DNA (0.01%) and proteins (1.76–6.60%). The two *L. mali* BR201 and FR123 strains produced 1.75 mg/mL and 1.95 mg/mL of EPS, respectively, and presented the same contaminations profile as the *Leuconostoc* EPS. They had a purity ~ 94%, and they were RNA free, with low contamination of DNA (< 0.01%) and proteins (~ 0.5%). After the purification steps, a recovery of ~ 69% was reached for the *L. mali* EPS and ranged from 89 to 98% for the *Lc. mesenteroides* EPS. The six biopolymers presented a purity of 99% to 100%. Therefore, these results support that growth in RAMGSR medium is suitable for high production of EPS by *L. mali* and *Lc. mesenteroides* strains with low contamination that can be removed by a simple purification method.

**Table 1:** Analysis of the EPS yield and their contaminants during purification

Samples	Supernatant^a^					After precipitation and dialysis^b^				
Strains	Biomolecules									
	EPS (mg/mL)	Protein^c^ (µg/mL)	DNA^c^ (ng/mL)	RNA^c^ (ng/mL)	EPS purity (%)	EPS (mg/mL)	Protein^c^ (µg/mL)	DNA^c^ (ng/mL)	RNA^c^ (ng/mL)	EPS purity (%)
*L. mali* BR201	1.75	106	62	< 20	94.28	1.19	< 1.0	< 0.5	< 20	100
*L. mali* FR123	1.95	115	123	< 20	94.42	1.37	< 1.0	25	< 20	99.99
*Lc. mesenteroides* A4X	3.90	205	73	< 20	93.39	3.52	18	19	< 20	99.49
*Lc. mesenteroides* Z36P	2.42	42	73	< 20	95.46	2.04	< 1.0	29	< 20	99.99
*Lc. mesenteroides* B12	2.65	38	27	< 20	98.58	2.35	< 1.0	< 0.5	< 20	100
*Lc. mesenteroides* O9	2.12	52	45	< 20	97.60	2.08	< 1.0	10	< 20	99.99

^a^Protein, DNA, and RNA concentrations were measured directly from cultures supernatants. EPS concentration was determined from neutral sugars estimation after ethanol precipitation from culture supernatants

^b^Solutions of the purified EPS were prepared in water at a concentration of 1 mg/mL

^c^The contaminants detection limits were 0.5 ng/mL for DNA, 20 ng/mL for RNA, and 1.0 µg/mL for proteins

**Fig. 5:**
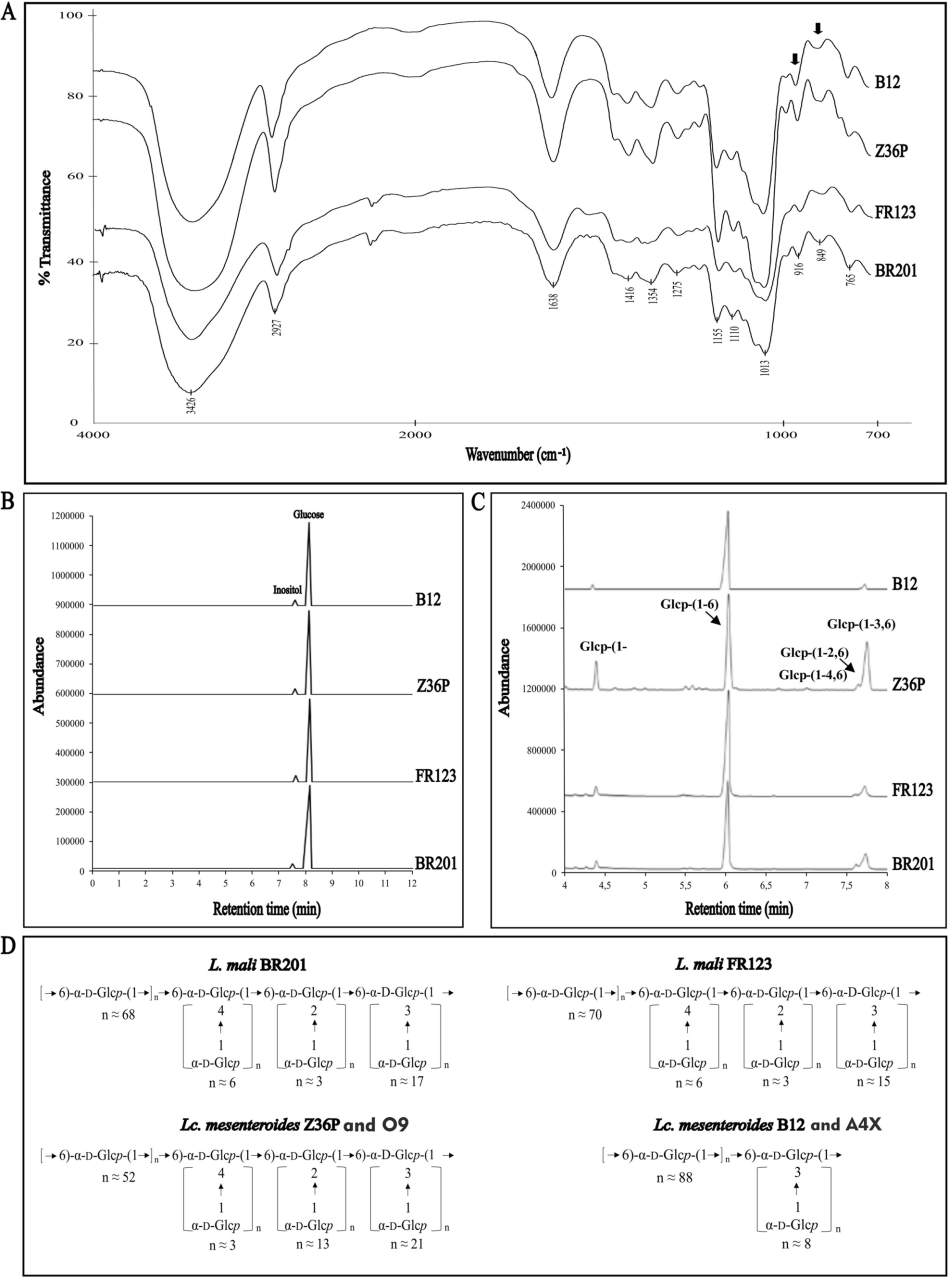
Structural characterization of EPS produced by *L. mali* BR201 and FR123, and *Lc. mesenteroides* Z36P and B12. Data from the *Lc. mesenteroides* 09 and AX4 EPS are not depicted in panels **A**, **B** and **C** for being the same as those of Z36P and B12, respectively. **A** Infrared spectra of the EPS, **B** EPS monomeric composition after hydrolysis, **C** EPS methylation analysis and **D** general structure of EPS. Structure of EPS from *Lc. mesenteroides* A4X and O9 are not shown, because there were identical to that of the EPS from *Lc. mesenteroides* B12 and Z36P, respectively

After purification, the six EPS were subjected to physicochemical characterization and in Fig. 5 are depicted the result obtained for *L. mali* BR201 and FR123 as well as *Lc. mesenteroides* B12 and Z36P. The analysis of the polymers from *Lc. mesenteroides* AX4 and O9 were not shown, because were the same as those obtained for B12 and Z36P, respectively. The results of the analysis of the monomeric composition demonstrate that all EPS were composed strictly of glucose units (Fig. 5B). In addition, the IR spectra of the EPS preparations of the *Leuconostoc* and the *Liquorilactobacillus* strains presented the same profile (Fig. 5A), typical of carbohydrates, with an absorption band between 848–853 cm^−1^ and 916–919 cm^−1^. Additionally, methylation analysis showed that all EPS had a main chain of glucopyranose units with (1,6) glycosidic linkages (between 63 and 93 units). Furthermore, two profiles were detected in the ramifications: (i) the EPS produced by *Lc. mesenteroides* B12 and A4X were partially branched in the *O*-3 position by glucopyranose units (10 and 8 units, respectively) (Fig. 5C), and (ii) the EPS produced by *Lc. mesenteroides* Z36P and O9 and *L. mali* BR201 and FR123 presented three ramification types (1,4), (1,2) and (1,3). The (1,3) and (1,2) ramifications showed, respectively, between 15 and 21, and between 3 and 13 units, these numbers being higher than those of the (1,4) type (between 3 and 6) (Fig. 5D).

### Quantification of EPS and riboflavin

To further investigate the behaviour of the Algerian LAB, the production of EPS in RAMGS and in RAMGSR by the six strains after 24 h of growth was quantified in cultures supernatants. The results are presented in Table 2.

**Table 2:** Quantification of EPS and riboflavin produced by LAB grown in the indicated media during 24 h

Strains	RAMGSR	RAMGS	
	EPS (g/L)^1^	EPS (g/L)^1^	Riboflavin (µg/L)^2^
*L. mali* BR201	2.12 ± 0.24^cd^	0.51 ± 0.04^e^	25.33 ± 1.40^b^
*L. mali* FR123	2.61 ± 0.23^bcd^	0.57 ± 0.03^e^	29.63 ± 1.08^b^
*Lc. mesenteroides* A4X	4.58 ± 0.29^a^	0.42 ± 0.03^e^	10.25 ± 1.62^b^
*Lc. mesenteroides* Z36P	2.63 ± 0.28^bc^	0.66 ± 0.05^e^	13.63 ± 0.83^b^
*Lc. mesenteroides* B12	2.67 ± 0.10^b^	2.48 ± 0.17^bcd^	230.46 ± 12.95^a^
*Lc. mesenteroides* O9	2.27 ± 0.12^bcd^	2.10 ± 0.23^d^	215.00 ± 15.83^a^

^1^EPS concentration in culture supernatants was determined after ethanol precipitation (1:3) by neutral sugars quantification by the phenol-sulfuric method

^2^Riboflavin fluorescence was measured in culture supernatants and its concentration was inferred from a riboflavin calibration curve

^a–e^Means with different letters differed significantly (*p* value ≤ 0.05)

Regarding to the riboflavin production, similar values were produced by *Lc. mesenteroides* B12 and 09 (~ 220 µg/L), approximately sixfold higher than that detected for *Lc. mesenteroides* A4X and Z36 (~ 12 µg/L). For *L. mali* strains, the production ranging from 25 and 29 µg/L for BR201 and FR123, respectively.

Concerning the EPS yield in RAMGSR and RAMGS (Table 2), in the presence of sucrose and riboflavin, A4X produced the highest EPS concentration (4.58 g/L), after incubating for 24 h. B12 and O9 showed the same level of production in RAMGSR and RAMGS (2.48 ± 0.17 g/L and 2.10 ± 0.23 g/L, respectively). By contrast, when riboflavin was not present in the medium, a drop of the EPS yield was observed for the other LAB belonging to either *Leuconostoc* or *Liquorilactobacillus* genus*,* with A4X **(**0.42 g/L) having the lowest value in RAMG.

### Influence of carbon source on LAB growth and metabolisms

**Fig. 6:**
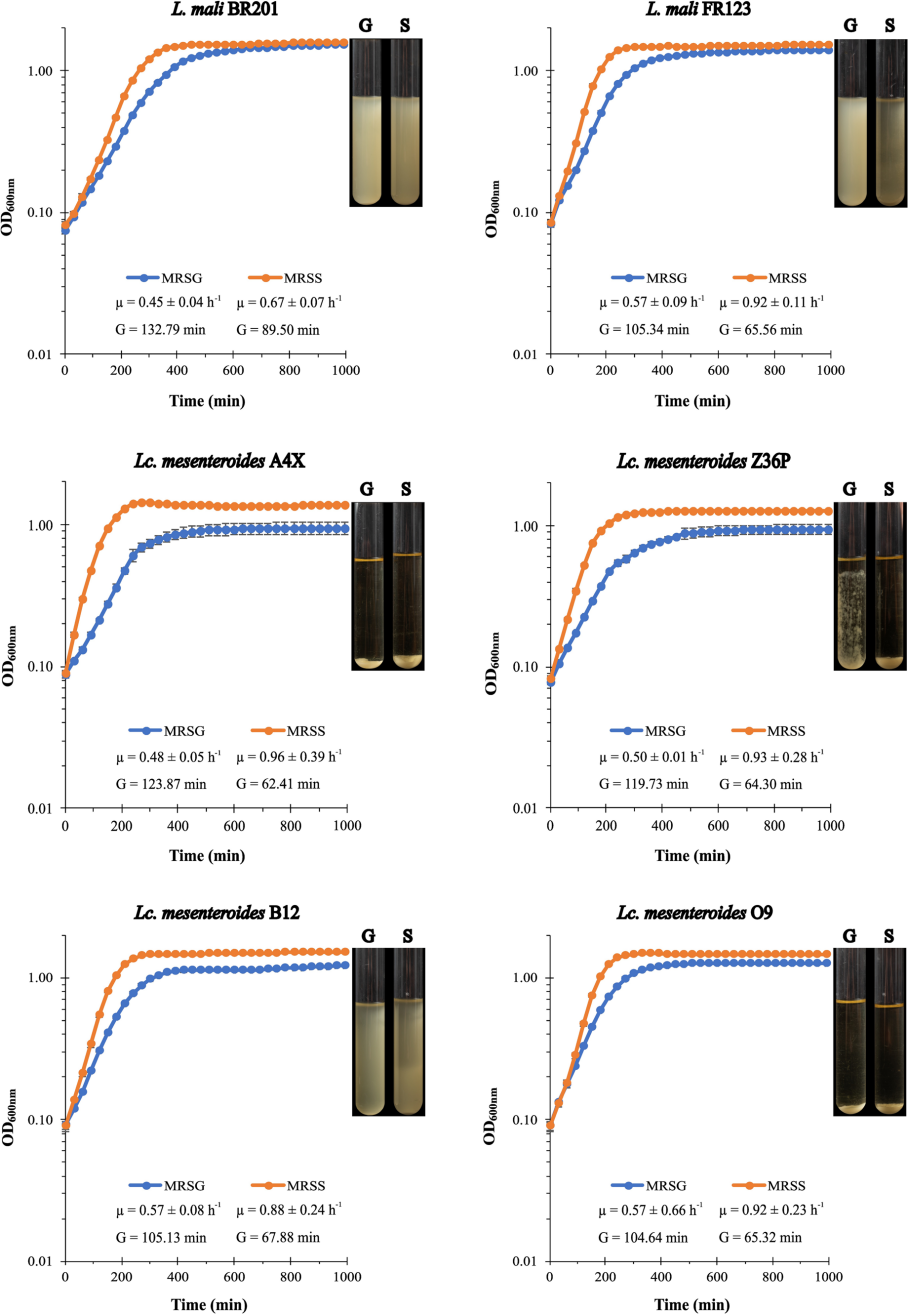
Influence of the carbon source on LAB growth. A comparative study, in real time, of LAB growth in MRSS and in MRSG. The growth rate (µ) as well as the generation time (G) of the cultures were calculated during the exponential phase. The values and standard deviation of three independent cultures are depicted

The Dsr catalyse the hydrolysis of the glycosidic bond of sucrose thereby generating the energy to catalyse the transfer of d-glucopyranosyl residues to the growing polymer (dextran) with a concomitant release of fructose. Moreover, there is not synthesis of the polymer in the absence of the substrate. Thus, to evaluate a possible beneficial effect of the Dsr action, the growth of the six strains in MRSS versus MRSG at 30 °C was compared and monitored in real time. For all strains, the presence of sucrose had a positive influence on the bacterial growth. In the case of the two lactobacilli, this was noted only at the exponential phase. Indeed, for *L. mali* strains, FR123 presented, on MRSS, a higher µ (0.92 h^−1^) and faster G (65 min) than BR201 (0.67 h^−1^, 89 min) and, consequently, a faster entry into the stationary phase (4.5 h vs. 6.5 h) (Fig. 6). The same profile was detected in the MRSG, FR123 grew faster than BR201 (0.57 h^−1^, 105 min vs. 0.45 h^−1^, 132 min). Both strains presented, at stationary phase, a similar final OD_600nm_ in MRSG and MRSS. However, the four *Lc. mesenteroides* strains grew well in the presence of the disaccharide at both phases, exponential and stationary. In MRSS A4X reached higher values of OD_600nm_ than in MRSG, prior to entry into the stationary phase (Fig. 6). Also, it grew faster in MRSS than in MRSG (G of 62 min vs. 123 min). The B12 and O9 strains showed the same µ and G in MRSG (0.57 h^−1^, 105 min), and Z36P and O9 shared almost the same growth parameters on MRSS (0.92 h^−1^, 65 min). Also, during the development of this work, it was observed that the six LAB presented differential growth characteristics in liquid media under static conditions (Insets in Fig. 6). The cells of the two *L. mali* strains and *Lc. mesenteroides* B12 appeared to be in suspension in both media tested. An opposite behaviour was observed for A4X and O9, whose cells were deposited at the bottom of the culture tubes upon growth in either MRSS or MRSG. By contrast, the Z36P cells in MRSG were stuck to the walls of the tubes.

Sugar consumption and metabolite secretion by cells can be measured in culture supernatants along with extracellular Dsr enzyme catalysis. Therefore, the influence of the Dsr catalysis in the general sugar metabolisms was tested analysing culture supernatants (Table 3), as well as the influence of sugar carbon source in the bacterial metabolic fluxes of the six LAB (Table 3, and Final OD of the cultures in Online Resource Table S5). When sucrose was the carbon source in the medium (116.8 mM) the four *Lc. mesenteroides* strains produced high concentration of mannitol (~ 12 g/L, ~ 72 mM) ranging from 59.91 mM for Z36P to 83.51 mM for O9 and only low concentration of lactic acid (10.63–13.91 mM). In the case of the *L. mali* strains, BR201 only produced 17.75 mM mannitol while FR123 generated 70.09 mM of this metabolite. Upon growth of BR201 in medium containing sucrose, it was still detected a concentration of sucrose of 7.78 mM and fructose accumulation (3.02 mM), not shown in the results. When, glucose was the carbon source in the medium, the six Algerian strains produced lactic acid, but secretion of mannitol was not detected. Addition of maltose to the media containing sucrose or glucose resulted in the production of other metabolites. In MRSGM, in addition to lactic acid production, ~ 0.20 mM maltotriose was generated by three strains (BR201, A4X and B12). In MRSSM, the six strains produced, in addition to high concentrations of mannitol, panose ranging from 16.63 mM for B12 to 37.53 mM for BR201.

**Table 3:** LAB metabolism using different carbon source

Strains	Medium	Final metabolites (mM)			
		Lactic acid	Mannitol	Maltotriose	Panose
*L. mali* BR201	MRSG	16.93 ± 0.17^de^	ND	ND	ND
	MRSGM	14.49 ± 0.08^ef^	ND	0.21 ± 0.01^A^	ND
	MRSS	20.27 ± 0.66^bc^	17.75 ± 0.39^q^	ND	ND
	MRSSM	18.03 ± 0.49^cd^	23.08 ± 0.27^p^	ND	37.53 ± 2.10^D^
*L. mali* FR123	MRSG	27.08 ± 0.82^a^	ND	ND	ND
	MRSGM	19.31 ± 0.09^ef^	ND	ND	ND
	MRSS	13.27 ± 0.34^fgh^	70.09 ± 0.89^kl^	ND	ND
	MRSSM	13.77 ± 0.19^fg^	67.38 ± 1.16^lm^	ND	17.83 ± 0.27^GH^
*Lc. mesenteroides* A4X	MRSG	19.82 ± 0.72^bc^	ND	ND	ND
	MRSGM	17.66 ± 0.50^cd^	ND	0.19 ± 0.005^ABC^	ND
	MRSS	12.83 ± 0.23^fgh^	71.23 ± 2.35^kl^	ND	ND
	MRSSM	12.54 ± 0.20^fgh^	63.80 ± 0.51^mn^	0.20 ± 0.01^A^	19.63 ± 0.15^F^
*Lc. mesenteroides* Z36P	MRSG	21.23 ± 0.35^b^	ND	ND	ND
	MRSGM	18.08 ± 0.62^cd^	ND	ND	ND
	MRSS	13.91 ± 2.30^f^	59.91 ± 5.02^n^	ND	ND
	MRSSM	9.54 ± 0.31^i^	49.93 ± 0.71^o^	0.17 ± 0.02^BC^	18.92 ± 0.18^FG^
*Lc. mesenteroides* B12	MRSG	17.66 ± 0.53^cd^	ND	ND	ND
	MRSGM	15.54 ± 0.07^def^	ND	0.20 ± 0.002^A^	ND
	MRSS	10.90 ± 0.68^hi^	72.84 ± 2.97^k^	ND	ND
	MRSSM	13.95 ± 0.34^f^	61.41 ± 0.04^mn^	0.16 ± 0.006^C^	16.63 ± 0.23^H^
*Lc. mesenteroides* O9	MRSG	21.64 ± 0.38^b^	ND	ND	ND
	MRSGM	18.02 ± 0.86^cd^	ND	ND	ND
	MRSS	10.63 ± 0.57^ghi^	83.51 ± 1.28^j^	ND	ND
	MRSSM	16.82 ± 0.04^de^	70.50 ± 0.33^kl^	ND	27.40 ± 0.5^E^

^a–i, j–q, A–C, D–H^Means with different letters differed significantly (*p* value ≤ 0.05). For not detected measurements (ND) a zero value was assumed

### Dextransucrase and dextranase activities

**Fig. 7:**
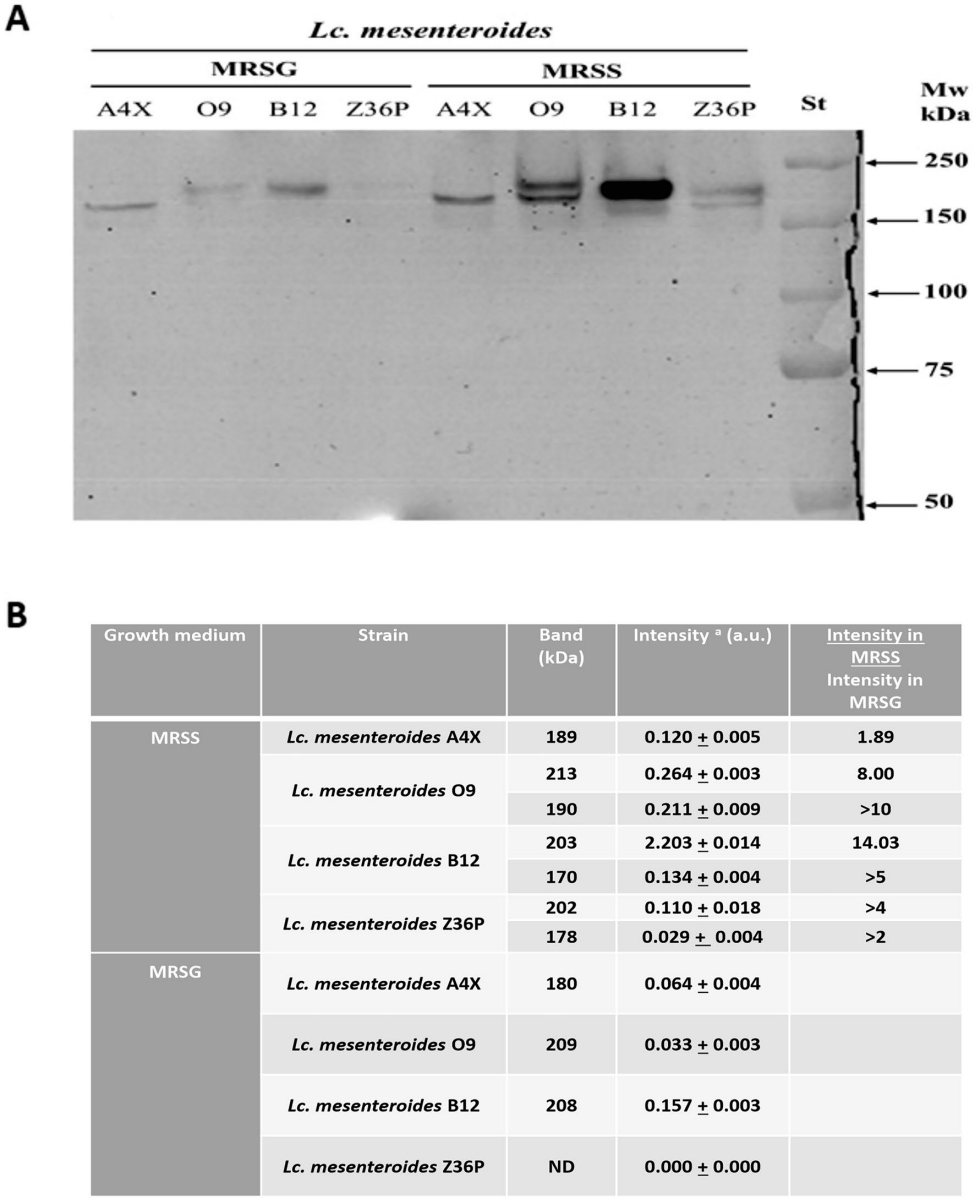
In situ detection of dextransucrase activity present in cultures’ supernatants of the *Leuconostoc mesenteroides* strains. The LAB were grown in presence of sucrose (MRSS) or glucose (MRSG), the cell free supernatants were exposed to SDS-PAGE and after protein renaturation were analyzed for enzymatic activity. **A** Zymogram. St, protein Mw standard. **B** Calibration curve made with protein standard and used to calculate Mw of the Dsr. **B** Table showing quantification of the intensity of the bands depicted in **A** and its estimated Mw. ^a^The limit of detection of intensity was 0.029 and 0.028 (a.u.) (for this figure, Fig. 8, respectively) . This detection limit was used to calculate the ratio of intensities (MRSS/MRSG), when the bands were not detected in supernatants of cultures grown in MRSG

**Fig. 8:**
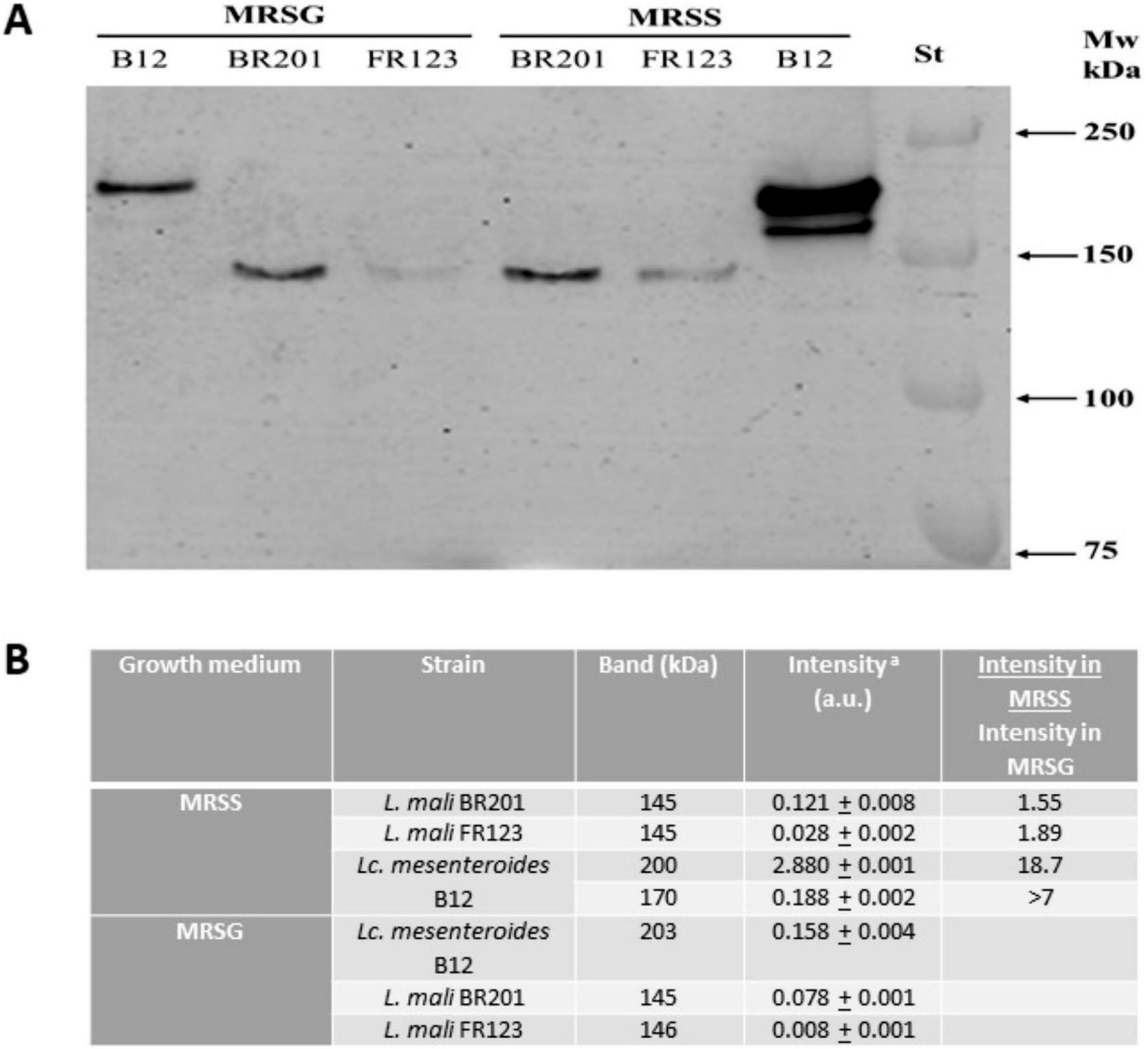
In situ detection of dextransucrase activity in culture supernatants of *Leuconostoc* and *Liquorilactobacillus* strains. The LAB were grown in presence of sucrose (MRSS) or glucose (MRSG), the cell free supernatants were exposed to SDS-PAGE and after protein renaturation were analyzed for enzymatic activity. **A** Zymogram. St, protein Mw standard. **B** Calibration curve made with protein standard and used to calculate Mw of the Dsr. **B** Table showing quantification of the intensity of the bands depicted in **A** and its estimated Mw. ^a^The limit of detection of intensity was 0.029 and 0.028 (a.u.) (for Fig. 7 and this figure, respectively). This detection limit was used to calculate the ratio of intensities (MRSS/MRSG), when the bands were not detected in supernatants of cultures grown in MRSG

In order to detect Dsr enzymes produced by the four *Lc. mesenteroides* strains and the two *L. mali* strains, responsible for the EPS production, supernatants of cultures grown in MRSG or MRSS were fractionated in SDS-polyacrylamide gels, and after removal of the detergent, the active Dsr were revealed by in situ synthesis of dextran, upon addition of sucrose, and Schiff staining. The obtained zymograms, in which the Dsr bands were visible, are depicted in Figs. 7A and 8A. The migration of the protein standard of known Mw was used to generate calibration curves (Fig. 1S), that were used to estimate the Mw of the Dsr (Figs. 7C, 8C). Moreover, in Tables presented in Figs. 7B and 8B detail the intensity of the bands. During the analysis, experimental variations in band migrations were recorded, which are related to the gels used and to the loading volume of supernatants.

In MRSS, the supernatants of *Lc. mesenteroides* O9, B12 and Z36P contain two active Dsr represented by two close bands (Fig. 7) with a Mw of 205 ± 5 kDa and 179 ± 10 kDa, while only the smaller Dsr was detected in the case of A4X. Concerning the detection of the Dsr in supernatants of the MRSG cultures, in the case of O9 and B12 the larger Dsr was also visualized, the smaller Dsr was only observed in the A4X sample, and no bands were detected in Z36P preparation. In addition, the intensity of some bands, quantified in Fig. 7C, revealed differences in the MRSS and MRSG culture supernatants (Fig. 7A). Thus, the following higher levels of active Dsr were detected in MRSS than in MRSG: (i) for A4X, ~ twofold of the 179 kDa; (ii) for O9, eightfold and > tenfold for the 205 kDa and the 179 kDa, respectively; (iii) for B12, fourteen-fold and > fivefold for the 205 kDa and the 179 kDa, respectively; (iv) for Z36P, > fourfold and > twofold for the 205 kDa and the 179 kDa enzymes. Consequently, for all *Lc. mesenteroides* strains the levels of all Dsr were higher in MRSS than in MRSG growth medium.

About the Dsr role of the Algerian *Lc. mesenteroides* strains, the physicochemical characterization of EPS produced by the LAB (Fig. 5) revealed that the strains having only one Dsr (A4X) or only one predominant band (B12) in MRSS (Fig. 7) produce EPS with a low percentage of ramification, whereas those showing two Dsr with similar intensity in MRSS (Z36P and O9) (Fig. 7) synthesize EPS with a high percentage of ramifications (Fig. 5).

For the two *Liquorilactobacillus* strains, only one Dsr of 145 ± 0.5 kDa was detected in MRSS and MRSG (Fig. 8). In this case, the levels of the active Dsr were similar in both media, only 1.5-fold and 1.9-fold higher in MRSS for the BR201 and FR123 strains, respectively.

**Fig. 9:**
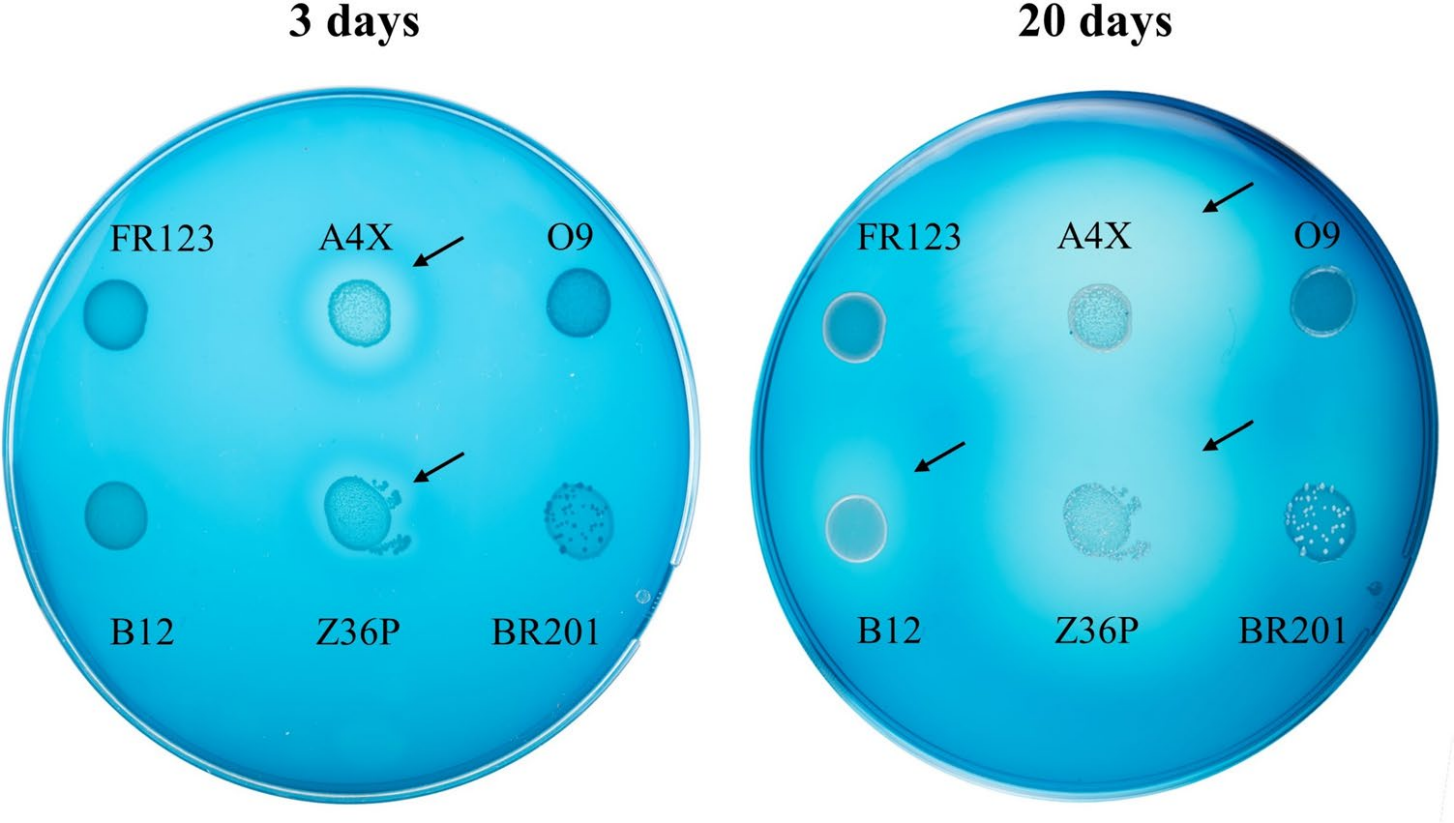
Detection of dextranase activity in LAB strains. Cultures of *Lc. mesenteroides* A4X, Z36P, B12 and O9 and *L. mali* BR201 and FR123 were spotted on MRSG-agar-blue dextran plates. Pictures were taken after incubation at 30 °C and after 3 and 20 days. The arrows indicate the halo of blue dextran degradation generated by A4X, Z36P and B12 strains.

For the detection of dextranase activity, only *Lc. mesenteroides* A4X, Z36P and B12 strains generated clear halos on MRSG-blue dextran-agar plates (Fig. 9) and relysed maltotriose to the culture supernatants in MRSSM (Table S5). After 3 days of incubation at 30 °C, the dextran degradation halos of A4X and Z36P strains began to appear and continued increasing up to 20 days (Fig. 9). During this incubation period, the appearance of a halo with weak activity was observed for the B12 strain. A strong activity represented by a large halo was detected in A4X and Z36P at the end of the incubation period.

## Discussion

In this work, we selected six LAB from different sources. A4X, FR123, and Z36P were isolated from the sap of the date palm, which is a turbid and sweet liquid extracted through the tapping method. The sap serves as an excellent natural refreshing beverage for the citizens of the Sahara, characterized by its good nutritional value. However, its consumption is limited in time and space as it transforms into an alcoholic beverage within a few hours at room temperature. O9 was isolated from freshly ground barley, which is receiving more attention from both agricultural and food scientists, because of its chemical composition and health benefits while B12 and BR201 were isolated from traditional Algerian dairy products: butter and sheep's milk. During the isolation step, we observed the abundance of exopolysaccharide-producing bacteria in the sap due to its sweet nature, which serves as a matrix for precursor substances (Makhlouf-Gafsi et al. 2016). By contrast, fresh barley proved to be the ecosystem with the lowest abundance of these types of bacteria which is closely tied to its chemical composition (Park et al. 2013).

The selection of the six LAB able to synthesize EPS was based on the use of sucrose as sugar in the medium which is the most used method to screen LAB strains producing homopolysaccharides (glucans, alternans and fructans) (Cirrincione et al. 2018). The comparison of different physiological and biochemical characteristics with the published taxonomic notes and species descriptions (Björkroth and Holzapfel 2006; Zheng et al. 2020) confirm the affiliation of strains to the species *L. mali* (BR201 and FR123) and *Lc. mesenteroides* (A4X, Z36P, O9 and B12). The strains identified were also confirmed by sequencing the partial variable of *rrs* and *pheS* genes data. According to Naser et al. (2007), who compared the sequence data of the *pheS* genes with the available *rrs* gene sequences, the use of the housekeeping genes such as *pheS* provided the highest discrimination for the identification of different species of lactobacilli. Several scientific works have previously described the presence of *Leuconostoc* and *Liquorilactobacillus* (previously *Lactobacillus*) species in traditional butter (Yu et al. 2018), sheep milk (Chen et al. 2020), date palm sap (Ziadi et al. 2011) and barley (Khumalo et al. 2022).

The six Algerian strains showed mucoid colonies only on MRSS- agar supplemented with sucrose, with different morphologies. This mucous sucrose-dependent phenotype has been previously observed for other strains belonging to the genera *Leuconostoc, Weissella* and *Lactobacillus* (Hernández-Alcántara et al. 2022; Llamas-Arriba et al. 2019; Notararigo et al. 2013; Shuai et al. 2023; Zarour et al. 2017).

Furthermore, TEM analysis did not reveal the presence of EPS in any strains cultured in MRSG, while in MRSS some EPS molecules surrounded the bacterial cells, and the majority were detached from the cells, as previously described for other *Leuconostoc* species and *Weissella* strains (Besrour-Aouam et al. 2019; Hernández-Alcántara et al. 2022; Zarour et al. 2017).

Cell biomass deposited at the bottom of the culture tube or on its wall may be related to the ability of the strain to form biofilms. Various studies indicated the capacity of LAB to aggregate and form biofilms which all support the use of probiotic biofilms producers LAB as alternative approach for reducing the formation of pathogenic biofilms in the food industries (Gómez et al. 2016; Tatsaporn and Kornkanok 2020). The biofilms from *Lactiplantibacillus plantarum* JCM1149 were resistant to environmental stress, which in turn can protect indirectly the bacterial cells (Kubota et al. 2008). This characteristic can be used as a safe method for combatting pathogenic growth in food and, consequently, improve public health (Mgomi et al. 2023; Werning et al. 2022). In the case of A4X, Z36P and O9 strains, the aggregation was observed not only in MRSG, but also in MRSS, which indicated that the presence of the EPS, generated from sucrose, does not affect negatively the bacterial aggregation. However, this is not a general behaviour, since previous results indicated that the presence of dextran prevented the aggregation of *L. sakei* MN1 cells (Nácher-Vázquez et al. 2017a) and, by contrast in the presence of the Dsr substrate, *Lc. lactis* AV1n and *Weissella cibaria* AV2ou formed biofilm more efficiently (Besrour-Aouam et al. 2021). In addition, it has also been proven that dextran produced by *L. hordei* MW 1.1822 is the main cause for inducing *S. cerevisiae* TMW 3.221 aggregation and network formation on hydrophilic surfaces, which is crucial for the initiation of the stepwise water kefir granule growth (Xu et al. 2018).

With regard to production of EPS by the LAB in liquid medium, we have previously shown that the chemically defined RAMGS medium was suitable for detection of EPS production by *Weissella* and *Leuconostoc* Spanish strains (Llamas-Arriba et al. 2021). The results presented here revealed that optimization and use of the synthetic medium RAMGSR allowed to recover EPS from *Lc. mesenteroides* and *L. mali* Algerian strains with high purity. The monomeric composition and IR spectra of purified EPS showed that they were glucans (Evans et al. 2020; Synytsya and Novak 2014) with α-configurations (Münkel et al. 2020). The additional analysis of methylation confirmed that the EPS produced by the six LAB were dextrans. The structures deduced for these polymers are depicted in Fig. 5C and coincide with those previously described (Besrour-Aouam et al. 2021; Yalmanci et al. 2022; Zarour et al. 2017) except the variety of branched chains in the dextrans of some strains: (1,3) in *Lc. mesenteroides* B12 and A4X, and different proportions of (1,2), (1,3) and (1,4) in *L. mali* BR201 and *L.* FR123 as well as in *Lc. mesenteroides* Z36P and O9. Recently, it has been shown that dextran produced by *Lc. mesenteroides* RSG7, isolated from cucumber, had α-(1,6) glycosidic linkages as backbone and α-(1,2), α-(1,3), α-(1,4) and α-(1,6) glycosidic linkages as side chains (Wang et al. 2023). Thus, it seems that *Lc. mesenteroides* dextrans with new types of branching are now being described.

Traditionally, the commercial RAMG medium has been used to select vitamin B_2_-producing bacteria based on their ability to grow in the absence of riboflavin. In this study, the result presented revealed that among the six LAB tested only B12 and 09 produced significant levels of riboflavin. Moreover, the increase of vitamin B_2_ concentration detected in cultures grown in RAMSR (results not shown) supports that there was not a detrimental feedback repression of the synthesis of this molecule by these LAB when the flavin is present in the environment, as previously observed for *L. plantarum* M5MA1-B2 (Mohedano et al. 2019), *Lactobacillus fermentum* PBCC11.5 (Russo et al. 2014), 8 *W. cibaria* strains (Diez-Ozaeta et al. 2023; Hernández-Alcántara et al. 2022) and *Leuconostoc citreum* BAL3C-4 and *Leuconostoc falkenbergence* (VSL11h-8 and VSL14h-1 strains) (Llamas-Arriba et al. 2021). In the case of the Algerian LAB tested here, for B12 and O9, the absence of riboflavin in the medium did not hinder their growth. Riboflavin is the precursor of the flavin mononucleotide and flavin dinonucleotide, and previous LAB reports showed that the ability to overproduce riboflavin is linked to the presence of the *rib* operon (Capozzi et al. 2012; Falasconi et al. 2020; Thakur et al. 2016). Thus, in the future it should be possible to increase the production of the two strains, by selecting of spontaneous mutants, as has been performed for other *Lc. mesenteroides* strains (Burgess et al. 2009).

The EPS quantification showed a high production rate, especially for *Lc. mesenteroides* A4X, in the presence of sucrose and riboflavin. The yield was higher than those produced by other *Leuconostoc* strains previously characterized by us, which varies between 1.25 and 3.14 g/L (Besrour-Aouam et al. 2019; Nácher-Vázquez et al. 2017a; Zarour et al. 2017) or other scientific works (Li et al. 2020; Wang et al. 2021a). It should be noted that the EPS yields are affected by the medium used, the concentration of sucrose, the incubation time (Yáñez-Fernández et al. 2021) and the method of isolation of the polymers (Siddharth et al. 2021).

The results of growth kinetics of the six LAB on liquid medium, in the presence of sucrose or glucose, showed that the use of sucrose, as carbon source, affected positively their growth, an effect that should allow them to reach higher biomass and to improve the bacterial metabolism. Also, the same effect was detected for strains belonging to the *Leuconostoc citreum*, *Leuconostoc falkenbergense* and *W. cibaria* genera upon growth in RAMS vs RAM (Llamas-Arriba et al. 2021). Studies of butterfly pea yogurt microbiota indicate the benefit of sucrose for LAB growth, resulting in a significant increase of its population and in a higher lactic acid production (Suharman et al. 2021).

Other studies showed that addition of sucrose improved the quality of silage fermentation (Wang et al. 2021b) and the probable intrinsic adaptation of sucrose-fermenting LAB to plant environments, where this substrate is abundant. These facts together with analysis of the influence of sucrose addition on carbohydrate metabolism and proteomic response of dextran-producing *Latilactobacillus sakei* and *Leuconostoc lactis* strains (Prechtl et al. 2018) support the use of these type of EPS-producing LAB as starters in sucrose-based food fermentations for the synthesis of dextran in situ.

The results of analysis of the effect of glucose, sucrose and maltose on the production of metabolites with functional and nutritional significance, revealed that the strains used in this study had the ability to produce in addition of lactic acid, the polyol mannitol, in the presence of sucrose and oligosaccharides: (i) maltotriose in the presence of glucose or sucrose plus maltose and (ii) its isomer, panose only in the presence of sucrose and maltose.

Previous studies indicated that in the presence of sucrose dextran-producing LAB belonging to the *Leuconostoc*, *Weissella* and *Latilactobacillus* genera release several functional metabolites such as fructose, mannitol and lactic acid (Besrour-Aouam et al. 2021; Nácher-Vázquez et al. 2017a; Zarour et al. 2018). Here, the metabolic fluxes detected in media containing sucrose revealed that the four *Lc. mesenteroides* and the *L. mali* FR123 strains upon sucrose hydrolysis by Dsr ~ 61% of the generated fructose is converted in mannitol by the action of the mannitol dehydrogenase, whereas the production of lactic acid by the phosphoketolase pathway was very inefficient (Martínez-Miranda et al. 2022).

When sucrose serves as the donor molecule and maltose as the acceptor molecule, the Dsr could catalyse the biosynthesis of glucooligosaccharides such as panose (Hanau et al. 2020). This trisaccharide is composed of a maltose molecule linked to a glucose molecule through an α-(1,6) glycosidic bond. This carbohydrate has potential applications as: (i) a functional oligosaccharide for the food industry, (ii) an anti-caries sweetener, (iii) anti-fading agent, (iv) antioxidant and (v) a prebiotic carbon source, because to its metabolization by a variety of probiotic bacteria (Ejby et al. 2016; Mäkeläinen et al. 2009; Wang et al. 2022a). Several studies have shown that panose production can be enhanced by adding maltose alongside sucrose. Koirala et al. (2019) detected panose after 24 h of fermentation of Brewers’ spent grain (containing maltose) supplemented with 4% of sucrose with *Lc. pseudomesenteroides* DSM20193 and *Weissella confusa* A16. In addition, it has been reported the production of 15 mM of panose by the Dsr of *L. hordei* TMW 1.1822 after 24 h of fermentation in MRS medium containing 100 mM of maltose and sucrose. Here, the Dsr from the LAB analysed produced in MRSSM from 16 to 37 mM, which seems to be very promising levels. Thus, the results presented here support the usage of the *Lc. mesenteroides* strains not only for the biofortification with the polymer but also with mannitol and panose.

The dextrans are enzymatically synthesized by extracellular Dsr using sucrose as substrate (Chen et al. 2021) and the zymogram analysis of culture supernatants performed here revealed that in the case of four *Lc. mesenteroides,* the levels of Dsr were induced, when sucrose was present in the growth medium. In addition, in all cases the levels of Dsr were drastically lower or undetectable, upon growth in MRSG. As an exception, in the case of A4X, where only the smaller Dsr was detected, only a 1.8-fold increase was detected in MRSS, indicating a synthesis of the enzyme almost constitutive. The majority of publications dealing with the detection in situ of Dsr in *Leuconostoc* species, such as *Lc. mesenteroides* and *Lc. lactis* showed that the synthesis of this enzyme is inducible in the presence of sucrose (Besrour-Aouam et al. 2021; Bounaix et al. 2010a; da Silva et al. 2019). Other species share the constitutive character such as *Streptococcus mutants* PR89 (Montville et al. 1978)*, Weissella* sp. (Bounaix et al. 2010b)*,*and, *W. cibaria* Av2ou and *W. confusa* FS54 (Besrour-Aouam et al. 2021).

With regard to the Mw, the majority of previous zymogram analysis of *Leuconostoc* Dsr showed appearance of one band with size of 200 kDa (Dawoud et al. 2021), 180 kDa (Bounaix et al. 2010a) and 170 kDa (Shukla and Goyal 2014). According to the complete protein sequences of the GH family 70 on NCBI Database, the highest Mw of extracellular *Lc. mesenteroides* Dsr ranged from 304 kDa (WP_182063986.1) to 299 kDa (WP_150289464.1). We propose that the appearance of multiple bands in some *Leuconostoc* strains may be related not only to the existance of various Dsr, but it could also be due to their ability to produce, through the glycoyltransferase (GTF) activity of various GH enzymes, a variety of polysaccharides from sucrose: (i) dextran and alternan by Dsr and alternansucrase (AS) (both belonging to the GH family 70), respectively, and (ii) fructans (levan) by levansucrases (LS) (GH family 68) (Nabot et al. 2022).

For the two *L. mali* strains, this is the first time that a zymogram analysis of the Dsr produced by strains of this species has been performed. In addition, analysis of the GH family 70 sequences of *L. mali* of 1055 amino acids, deposited in the NCBI databases, revealed a Mw estimated as 112.9 kDa (WP_056990607.1), thus being apparently smaller than those detected by us of 145 kDa.

Furthermore, GTF activity of Dsr has been detected for lactobacilli in zymograms, after incubation in sucrose buffer, showing single or several activity bands at approximately 148 kDa for *L. plantarum* DM5 (Das and Goyal 2014) and Dsr around 130 kDa from *L. hordei* TMW 1.1822 (Schmid et al. 2019). We have also previously characterized the *L. sakei* MN1 *dsrLS* gene of 5304 bp, which encodes the DsrLS composed of 1767 amino acids (ATN28243.1) corresponding to an extracellular Dsr of 178 kDa (Nácher-Vázquez et al. 2017b). Thus, in the case of *L. mali* strains as in other lactobacilli Dsr have been detected*.*

In this work, we have also detected a dextranase activity in three strains of *Lc. mesenteroides*. These results do not correlate with the levels of dextran obtained after quantification, because the EPS were produced during 24 h of incubation and the activity of the dextranase only appeared after 3 days of incubation. The relationship between the dextranase activity and the decrease in dextran levels reflecting its degradation has been suggested for the characterization of *W. confusa* FS54 dextranase producing strain (Besrour-Aouam et al. 2021). On the other hand, a close relationship was observed between the mucoid phenotype of the colonies of these three (A4X, Z36P and B12) strains and the degrading dextran activity. The strains presenting a large halo, A4X and Z36P, gave on the MRSS-agar flat colonies with degraded appearance, while the B12 strain, with a medium dextranase halo, gave a colony as gel diffused on medium. Furthermore, apart from *S. mutants* ATCC 25175 (Suzuki et al. 2012) and *Streptococus creciti* E49 (Tamura et al. 2007) and the work cited above on *W. confusa* FS54, no natural dextranase activity has been described in any LAB (Zannini et al. 2016). As far as we know, this is the first instance of a dextranase activity in strains belonging to the *Leuconostoc* genus.

In summary, the six LAB isolated during this study and characterized for their production of significant postbiotics (EPS, mannitol, vitamin B_2_, and prebiotics), can enhance the nutritional composition and improve efficiently the technological and rheological properties of their ecosystems to be used as healthy and functional foods.

## Conclusions

Six LAB strains isolated from dairy and vegetable products have been characterized in this work. The strains belonging to *Lc. mesenteroides* (4) and *L. mali* (2) species were identified as dextran and oligosaccharide producing bacteria. All the dextrans have (1,6) glycosidic linked glucose subunits in the main chain, but differ in the type and location of the branches. The key enzymes for dextran production, dextransucrases, have been detected in situ, and they have different molecular weights*.* All bacteria grew better in presence of sucrose than in glucose. All strains catalysed an efficient production of mannitol, when sucrose was the carbon source. In addition, production of panose required the presence of fructose and maltose in the growth medium. Moreover, out of the four *L. mesenteroides* strains, three (A4X, Z36P and B12) have dextranase activity and, two (B12 and O9) were able to produce significant amounts of vitamin B_2_. Therefore, the metabolic versatility of the LAB strains isolated in this work opens up several lines of research to acquire new knowledge to design new enriched products for the competitive market of functional foods.

### Supplementary Information

Below is the link to the electronic supplementary material.

Supplementary file1 (DOCX 144 KB)

Supplementary file2 (DOCX 32 KB)

## Data Availability

The datasets generated during and/or analysed during the current study are available from the corresponding author on reasonable request.

## Abbreviations

Dsr: Dextransucrases
EPS: Exopolysaccharide
G: Generation time
GC–MS: Gas chromatography–mass spectrometry
LAB: Lactic acid bacteria
µ: Growth rate
MRSG: Man Rogosa Sharpe broth containing 2% glucose
MRSS: Man Rogosa Sharpe broth containing 2% sucrose
NCBI: National Center or Biotechnology Information
RAMGS: Riboflavin Assay Medium supplemented with 2% of glucose and 2% of sucrose
RAMGSR: RAMGS supplemented with riboflavin (1 µg/mL)
TEM: Transmission electron microscopy
