# Supplementary material for: Leuconostoc mesenteroides and Liquorilactobacillus mali strains, isolated from Algerian food products, are producers of the postbiotic compounds dextran, oligosaccharides and mannitol
Source: World J Microbiol Biotechnol. 2024 Feb 29;40(4):114. doi: 10.1007/s11274-024-03913-3 (PMC10901973; doi:10.1007/s11274-024-03913-3)
Supplement: Supplementary file 1 — Supplementary file1 (DOCX 144 KB) [file 11274_2024_3913_MOESM1_ESM.docx]

***Leuconostoc mesenteroides* and *Liquorilactobacillus mali* strains, isolated from Algerian food products, are producers of the postbiotic compounds dextran, oligosaccharides and mannitol**

**Kenza Zarour^a,b^ (ORCID:** **0000-0002-5893-3343), Ahmed Fouad Zeid^a,b^ (ORCID:** **0000-0003-3594-2324), Mari Luz Mohedano^a^ (ORCID: 0000-0001-6748-9443), Alicia Prieto^a^ (ORCID: 0000-0002-5075-4025), Mebrouk Kihal^b^ (ORCID: 0000-0003-2901-373X) and Paloma López^a,*^ (ORCID: 0000-0001-8755-8952)**

^a^Departamento de Biotecnología Microbiana y de Plantas, Centro de Investigaciones Biológicas Margarita Salas (CIB, CSIC), 28040 Madrid, Spain

^b^Laboratoire de Microbiologie Appliquée. Faculté des Sciences de la Nature et de la Vie, Université Oran 1 Ahmed Ben Bella, Es Senia. 31100 Oran, Algeria.

*Corresponding author. Tel.: +34 918373112 Ext. 4202; Fax: +34 915360432. E-mail address: [plg@cib.csic.es](mailto:plg@cib.csic.es) (P. López).

**Figure S1**


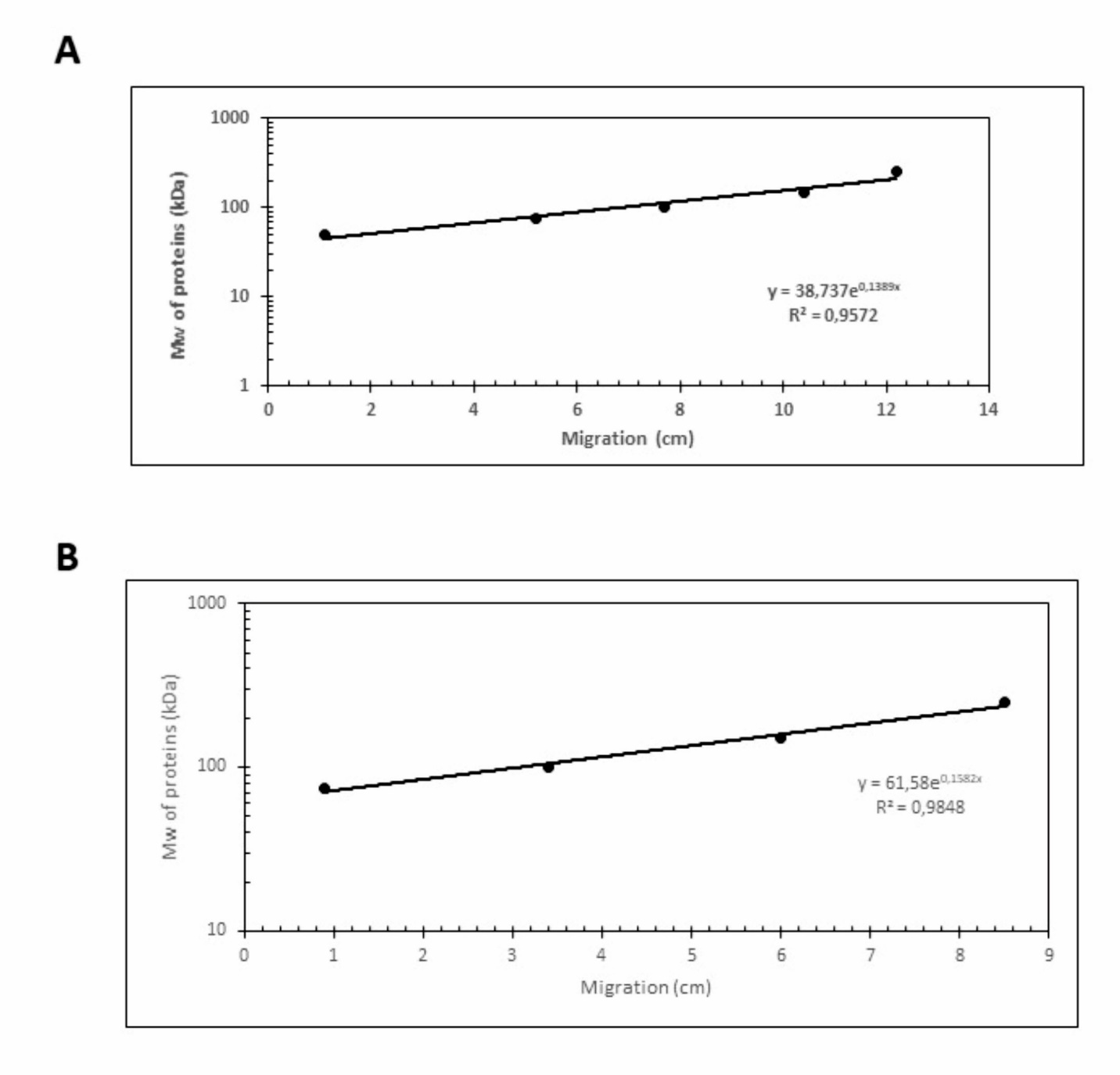


**Figure S1.** Calibration curves made with protein standard and used to calculate Mw of the Dsr detected in the zymograms depicted in Figure 7 (A) and Figure 8 (B).
