# Supplementary material for: Leuconostoc mesenteroides and Liquorilactobacillus mali strains, isolated from Algerian food products, are producers of the postbiotic compounds dextran, oligosaccharides and mannitol
Source: World J Microbiol Biotechnol. 2024 Feb 29;40(4):114. doi: 10.1007/s11274-024-03913-3 (PMC10901973; doi:10.1007/s11274-024-03913-3)
Supplement: Supplementary file 2 — Supplementary file2 (DOCX 32 KB) [file 11274_2024_3913_MOESM2_ESM.docx]

***Leuconostoc mesenteroides*****and *Liquorilactobacillus mali* strains, isolated from Algerian food products, are producers of the postbiotic compounds dextran, oligosaccharides and mannitol**

**Kenza Zarour^a,b^ (ORCID:** **0000-0002-5893-3343), Ahmed Fouad Zeid^a,b^ (ORCID:** **0000-0003-3594-2324), Mari Luz Mohedano^a^ (ORCID: 0000-0001-6748-9443), Alicia Prieto^a^ (ORCID: 0000-0002-5075-4025), Mebrouk Kihal^b^ (ORCID: 0000-0003-2901-373X) and Paloma López^a,*^ (ORCID: 0000-0001-8755-8952)**

^a^Departamento de Biotecnología Microbiana y de Plantas, Centro de Investigaciones Biológicas Margarita Salas (CIB, CSIC), 28040 Madrid, Spain

^b^Laboratoire de Microbiologie Appliquée. Faculté des Sciences de la Nature et de la Vie, Université Oran 1 Ahmed Ben Bella, Es Senia. 31100 Oran, Algeria.

*Corresponding author. Tel.: +34 918373112 Ext. 4202; Fax: +34 915360432. E-mail address: [plg@cib.csic.es](mailto:plg@cib.csic.es) (P. López).

**Table S1** Physiological and biochemical characteristics of LAB strains

| Growth conditions | | | | | | | |  |  |  |  |  |  |
| --- | --- | --- | --- | --- | --- | --- | --- | --- | --- | --- | --- | --- | --- |
| - Tests   Strain | **EPS production in MRSS** | **CO_2_ production in MRSG** | **Arginine dihydrolase**  **activity** | **Citrate metabolism** | **Acetoin production** | **Temperature (ºC)** | | | | **NaCl (%)** | | **pH** | |
|  |  |  |  |  |  | **4** | **15** | **37** | **45** | **3.0** | **6.5** | **4.0** | **8.0** |
| *L. mali* BR201 | + | - | - | + | + | - | + | + | - | + | + | + | - |
| *L. mali* FR123 | + | - | - | + | + | - | + | + | - | + | + | + | - |
| *Lc. mesenteroides* A4X | + | + | - | + | + | - | + | + | - | + | + | + | - |
| *Lc. mesenteroides* Z36P | + | + | - | + | - | - | + | + | - | + | - | + | - |
| *Lc. mesenteroides* B12 | + | + | - | + | - | - | + | + | - | + | - | + | - |
| *Lc. mesenteroides* O9 | + | + | - | + | - | - | + | + | - | + | - | + | - |

**Table S2** Test of the LAB strains for carbohydrates metabolism with AP1 50 CHL kit

| Carbon source | *L. mali* | | *Lc. mesenteroides* | | | |
| --- | --- | --- | --- | --- | --- | --- |
|  | **BR201** | **FR123** | **A4X** | **Z36P** | **B12** | **O9** |
| L-arabinose | - | - | + | + | - | - |
| D-xylose | - | - | + | + | + | + |
| D-galactose | + | - | + | + | + | + |
| D-glucose | + | + | + | + | + | + |
| D-fructose | + | + | + | + | + | + |
| D-mannose | + | + | + | + | + | + |
| L-sorbose | - | - | + | + | - | - |
| Methyl-αD-Glucopyranoside | - | - | + | + | + | + |
| N-acetylglucosamine | + | + | + | + | + | + |
| Amygdalin | + | + | - | - | - | - |
| Arbutin | + | + | - | - | - | - |
| Esculin | + | + | + | + | + | + |
| D-cellobiose | + | + | - | - | - | - |
| D-maltose | - | - | + | + | + | + |
| D-lactose | + | - | + | + | + | + |
| D-melibiose | - | - | + | + | + | + |
| D-sucrose | + | + | + | + | + | + |
| D- trehalose | + | + | + | + | + | + |
| D-raffinose | - | - | + | + | + | + |
| Gentiobiose | + | + | + | + | + | + |
| D-turanose | - | - | + | + | + | + |
| D-tagatose | + | + | - | - | - | - |

**Table S3** Bacteria used to develop the tree of the 16S rRNA coding genes presented in Fig.1.

| Strain | Habitat | Country of isolation |
| --- | --- | --- |
| *Leuconostoc mesenteroides* subsp. *dextranicum* DSM 20484 (CP012009.1) | Cheese, Type strain | South Korea |
| *Leuconostoc mesenteroides* subsp. *mesenteroides* J18 (CP003101.3) | kimchi | South Korea |
| O9 (OQ536305) | Barely | Algeria |
| B12 (OQ536302) | Traditional butter | Algeria |
| Z36P (OQ536304) | Date palm sap | Algeria |
| A4X (OQ536298) | Date palm sap | Algeria |
| *Leuconostoc mesenteroides* subsp*. jonggajibkimchii* DRC1506 (CP014611.1) | Kimchi | South Korea |
| *Leuconostoc mesenteroides* SRCM102735 (CP028255.1) | Soybean paste (Chonggugjang) | South Korea |
| *Leuconostoc suionicum* LT-38 (AP017935.1) | ND | Japan |
| *Leuconostoc pseudomesenteroides* CBA3630 (CP042383.1) | kimchi | South Korea |
| *Leuconostoc lactis* CBA3626 (NZ_CP042387.1) | kimchi | South Korea |
| *Leuconostoc gasicomitatum* LMG 18811 (NC_014319.1) | Marinated broiler meat | Finland |
| *Leuconostoc carnosum* CBA3620 (NZ_CP042374.1) | kimchi | South Korea |
| *Limosilactobacillus reuteri* M2021619 (CP090476.1) | Fecal sample from healthy young woman | China |
| *Liquorilactobacillus nagelii* TMW 1.1827 (CP018180.1) | Water kefir | Germany |
| *Pediococcus pentosaceus* GDIAS001 (CP046938.1) | Plant feed material-topioca | China |
| BR201 (OQ538136) | Sheep milk | Algeria |
| FR123 (OQ536309) | Date palm sap | Algeria |
| *Liquorilactobacillus mali* LM596 (CP045035.1) | Apple juice from cider press | South Korea |
| *Liquorilactobacillus hordei* TMW 1.1822 (CP018176.1) | Water kefir | Germany |
| *Liquorilactobacillus hordei* DSM 19519 (CP049303.1) | Malted barley | Belgium |
| *Liquorilactobacillus nagelii* DSM 13675 (CP049304.1) | Partially fermented wine | USA |
| *Bacillus subtilis subsp. spizizenii* ATCC 6633 (NZ_CP034943.1) | Type strain | ND |

**Table S4** Bacteria used to develop the tree of the *pheS* genes presented in Fig. 2.

| Strains | Habitat | Country |
| --- | --- | --- |
| *Leuconostoc mesenteroides* subsp. *jonggajibkimchii* DRC1506 (CP014611.1) | Kimchi | South Korea |
| *Leuconostoc mesenteroides* SRCM102735 (CP028255.1) | Soybean paste (Chonggugjang) | South Korea |
| B12 (OQ592381) | Traditional butter | Algeria |
| *Leuconostoc mesenteroides subsp. mesenteroides* J18 (CP003101.3) | kimchi | South Korea |
| *Leuconostoc mesenteroides* subsp. *dextranicum* DSM 20484 (CP012009.1) | Cheese. Type strain | South Korea |
| *Leuconostoc suionicum* LT-38 (AP017935.1) | ND | Japan |
| Z36P (OQ592382) | Date palm sap | Algeria |
| A4X (OQ592380) | Date palm sap | Algeria |
| *Leuconostoc carnosum* CBA3620 (NZ_CP042374.1) | kimchi | South Korea |
| *Leuconostoc lactis* CBA3626 (NZ_CP042387.1) | kimchi | South Korea |
| *Leuconostoc gasicomitatum* LMG 18811 (NC_014319.1) | Marinated broiler meat | Finland |
| *Leuconostoc pseudomesenteroides* CBA3630 (CP042383.1) | kimchi | South Korea |
| *Liquorilactobacillus nagelii* TMW 1.1827 (CP018180.1) | Water kefir | Germany |
| *Liquorilactobacillus nagelii* DSM 13675 (CP049304.1) | Partially fermented wine | USA |
| *Liquorilactobacillus hordei* DSM 19519 (CP049303.1) | Malted barley | Belgium |
| *Liquorilactobacillus hordei* TMW 1.1822 (CP018176.1) | Water kefir | Germany |
| BR201 (OQ592384) | Sheep milk | Algeria |
| FR123 (OQ592383) | Date palm sap | Algeria |
| *Liquorilactobacillus mali* LM596 (CP045035.1) | Apple juice from cider press | South Korea |
| *Pediococcus pentosaceus* GDIAS001 (CP046938.1) | Plant feed material-topioca | China |
| *Limosilactobacillus reuteri* M2021619 (CP090476.1) | Fecal sample from healthy young woman | China |
| *Bacillus subtilis* subsp. spizizenii ATCC 6633 (NZ_CP034943.1) | Type strain | ND |

**Table S5** Final OD_600 nm_ of LAB cultures grown at 30 ºC in the indicated media.

| **Strains** | **Media** | **Final OD** |
| --- | --- | --- |
| *L. mali* BR201 | MRSG | **2.90** |
|  | MRSG M | **2.27** |
|  | MRSS | **6.83** |
|  | MRSSM | **3.97** |
| *L. mali* FR123 | MRSG | **3.43** |
|  | MRSGM | **2.57** |
|  | MRSS | **2.23** |
|  | MRSSM | **1.01** |
| *Lc. mesenteroides* A4X | MRSG | **2.70** |
|  | MRSGM | **2.23** |
|  | MRSS | **7.73** |
|  | MRSSM | **3.57** |
| *Lc. mesenteroides* Z36P | MRSG | **1.97** |
|  | MRSGM | **2.17** |
|  | MRSS | **5.63** |
|  | MRSSM | **4.33** |
| *Lc. mesenteroides* B12 | MRSG | **2.47** |
|  | MRSGM | **1.97** |
|  | MRSS | **4.43** |
|  | MRSSM | **3.97** |
| *Lc. mesenteroides* O9 | MRSG | **2.50** |
|  | MRSGM | **2.03** |
|  | MRSS | **5.97** |
|  | MRSSM | **3.60** |

The supernatants of these cultures were used to detect sugars and metabolites concentrations by GC-MS (Table 3). The bacteria were grown for 24 h. Beside *L. mali* FR123, which was grown until reached similar OD_600 nm_ than the other five LAB.
